# Dehydroglutathione, a glutathione derivative to introduce non-reversible glutathionylation†

**DOI:** 10.1039/d5cb00052a

**Published:** 2025-05-21

**Authors:** Daniel Oppong, Rayavarapu Padmavathi, Dhanushika S. K. Kukulage, Madhu C. Shivamadhu, Elizabeth A. Newberry, Anneliese M. Faustino, Hsin-Yao Tang, Young-Hoon Ahn

**Affiliations:** a Department of Chemistry, Drexel University Philadelphia PA 19104 USA ya426@drexel.edu +1 (215) 895-2666; b The Wistar Institute Philadelphia PA 19104 USA

## Abstract

Protein cysteine is susceptible to diverse oxidations, including disulfide, *S*-sulfenylation, *S*-nitrosylation, and *S*-glutathionylation, that regulate many biological processes in physiology and diseases. Despite evidence supporting distinct biological outcomes of individual cysteine oxoforms, the approach for examining functional effects resulting from a specific cysteine oxoform, such as *S*-glutathionylation, remains limited. In this report, we devised a dehydroglutathione (dhG)-mediated strategy, named G-PROV, that introduces a non-reducible glutathionylation mimic to the protein with the subsequent delivery of the modified protein to cells to examine the “phenotype” attributed to “glutathionylation”. We applied our strategy to fatty acid binding protein 5 (FABP5), demonstrating that dhG induces selective modification at C127 of FABP5, resembling *S*-glutathionylation. dhG-modified glutathionylation in FABP5 increases its binding affinity to linoleic acid, enhances its translocation to the nucleus for activating PPARβ/δ, and promotes MCF7 cell migration in response to linoleic acid. Our data report a facile chemical tool to introduce a glutathionylation mimic to proteins for functional analysis of protein glutathionylation.

## Abstract 2

A glutathione-derived chemical tool was developed to mimic glutathionylation in proteins, demonstrating that the glutathione modification of FABP5 cysteine 127 induces cancer cell migration.

## Graphical abstract

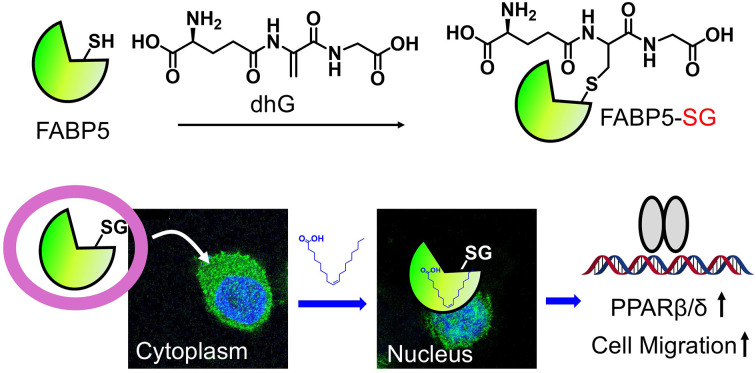

## Introduction

Cysteine in proteins is unique for its high nucleophilicity and oxidation-susceptibility.^1,2^ These features allow protein cysteines to exert distinct biological functions, such as redox sensing.^1,3^ Cysteine oxidations occur in response to diverse biological, medical, and environmental factors, including growth factors and cytokines,^4–6^ reactive oxygen species (ROS),^7^ nutrients,^8–10^ chemotherapy,^11,12^ radiation,^13^ and metals,^14,15^ suggesting their prevalence and significance in biology and medicine. The complexity of cysteine oxidations is that cysteine forms diverse oxoforms, including *S*-sulfenylation, disulfide formation, *S*-glutathionylation, and *S*-nitrosylation, which retain unique structures and chemical reactivity.^3^ Although different cysteine oxoforms could cause similar functional outcomes (*e.g.*, inhibiting enzyme activity), evidence supports that individual cysteine oxidations occur on distinct sets of proteins and regulate different biological pathways and processes,^16^ warranting investigation of individual cysteine oxidations.

Protein *S*-glutathionylation is one of the main cysteine oxidations and represents protein cysteine disulfide bond formation with intracellular glutathione.^17^ The significance of protein *S*-glutathionylation has been demonstrated through its regulatory or contributing roles in physiology and pathology,^17,18^ including proliferation, migration,^4,19^ inflammation,^20^ fibrosis,^21^ the cardiovascular system,^22,23^ neurodegeneration,^24^ and cancers,^25^ among others. It is notable that the development of biochemical tools and strategies, especially in conjugation with proteomics and mass spectrometry, has enabled the identification of a large number of proteins and cysteines (*n* > 2000) susceptible to *S*-glutathionylation.^17^ The cysteine sites identified *via* proteomics serve as important candidates for uncovering biological functions upon their glutathionylation.^4^ However, the general strategy for functional analyses relies on comparing biological phenotypes between two cohorts of cells expressing wild-type (WT) or Cys mutants (*e.g.*, Cys to Ser or Ala) of a protein of interest (POI), *i.e.*, mutating an oxidizable Cys residue.^4,26,27^ Although effective, this approach concludes that biological functions are attributed to the POI's “oxidation” *per se*, while there is a lack of direct evidence linking the POI's “glutathionylation” to biological phenotypes.

**Fig. 1:**
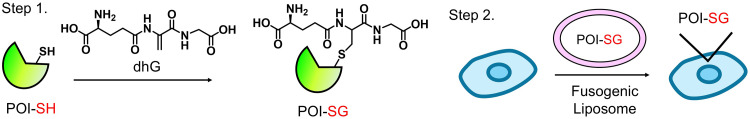
G-PROV approach for functional study of glutathionylated proteins in cells. The protein of interest (POI) is subjected to a reaction with dehydroglutathione (dhG) *in vitro*, which induces a non-reducible mimic of glutathionylation (step 1). The dhG-mediated glutathione-modified POI is delivered to the cytoplasm of cells *via* a fusogenic liposome for functional phenotype analysis (step 2).

To address this limitation, we devised a strategy in this report, named “dehydroglutathione (dhG)-induced protein glutathionylation and delivery” (G-PROV) (Fig. 1). The strategy involves two steps: (1) introducing a non-reducible glutathionylation mimic onto POI using dhG (step 1) and (2) delivery of the modified POI to cells (step 2), where the functional effects of POI with a glutathionylation mimic can be investigated. We applied our strategy to fatty acid binding protein 5 (FABP5). FABP5 is one FABP isoform that plays an important role in lipid transport and metabolism.^28^ FABP5 is implicated in metabolic syndrome, neurologic diseases, inflammation, and cancers.^28,29^ Previous studies showed that FABP5 is a redox-active protein, forming intracellular disulfide and *S*-glutathionylation.^30–32^ Functional analysis demonstrated that FABP5 *S*-glutathionylation suppresses lipopolysaccharide-induced inflammation in macrophages.^31^ In this study, we showed that the G-PROV strategy induces a glutathione modification in FABP5, resembling *S*-glutathionylation. We demonstrated that dhG-derived glutathione modification in FABP5 increases its binding affinity with linoleic acid (LA), activates peroxisome proliferator-activated receptor β/δ (PPARβ/δ), and increases migration of MCF7 cells upon incubation of LA. Our report provides a new facile strategy for the functional study of protein glutathionylation, while providing evidence linking “FABP5 glutathionylation” to “cancer cell migration.”

## Results

### dhG generates a glutathione modification on Cys resembling glutathionylation

dhG contains dehydroalanine (dhA) instead of l-Cys in glutathione (γGlu-Cys-Gly).^33^ The Michael acceptor in dhG can react with protein cysteine to form a protein–glutathione conjugate with a thioether linkage (P-SG) in place of disulfide (PS-SG) in glutathionylation. While P-SG differs from PS-SG in that it lacks one sulfur atom and likely loses stereochemistry at Cys in glutathione, we envisioned it to be a close mimic of glutathionylation and non-reducible, and it may induce similar functional effects to glutathionylation.

**Fig. 2:**
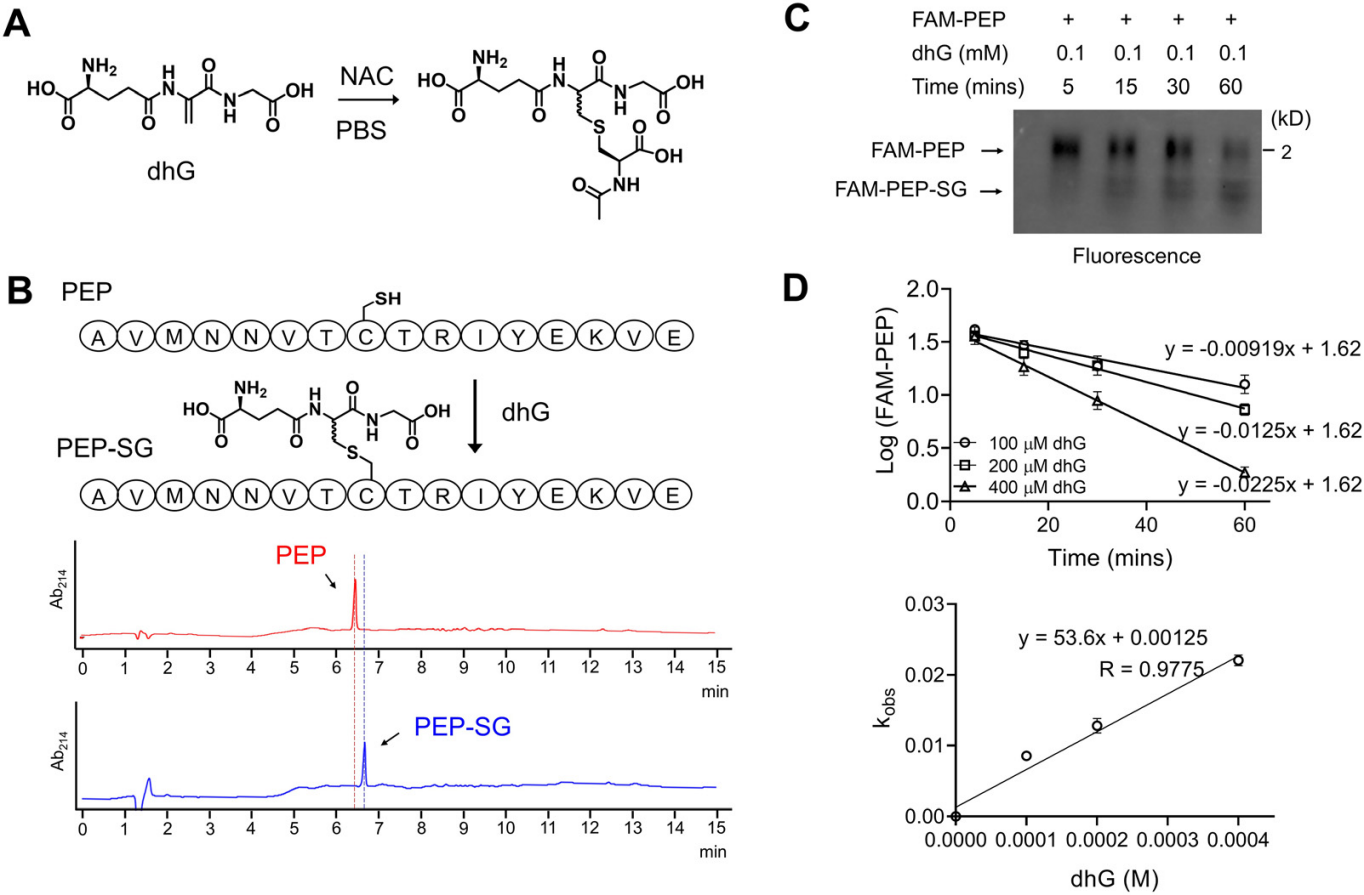
dhG reaction with Cys results in a thioether linkage of glutathione modification. (A) dhG reaction with *N*-acetylcysteine (NAC), resulting in the Michael reaction adduct. (B) The reaction of a Cys-containing peptide (PEP) with dhG. PEP (0.1 mM) was incubated without (top) or with dhG (1 mM) (bottom) in PBS (pH 8) for 1 h, and was analyzed by HPLC-MS (monitoring absorbance at 214 nm). (C) The reaction of fluorescein-conjugated PEP (FAM-PEP) with dhG in urea-gel electrophoresis (*n* = 3, biological replicates). FAM-PEP and its conjugation product were monitored by fluorescence. (D) dhG reaction kinetics with FAM-PEP. The intensities of the FAM-PEP bands at different times in urea-gels were plotted for the rate (top). Reactions were assumed to follow pseudo-first-order kinetics. Reaction rates were plotted as a function of dhG concentration (bottom) to determine the second-order rate constant (*n* = 3, biological replicates). Data show the mean ± SD (D) and are representative of replicate experiments (B) and (C).

dhG was synthesized in two steps from glutathione (Fig. S1A, ESI†).^33^ dhG was then tested for its reaction with cysteine. The incubation of dhG with *N*-acetylcysteine (NAC) in PBS resulted in the Michael reaction product containing the thioether bond (Fig. 2A), confirmed by NMR (Fig. S1B, ESI†). Next, dhG was examined for its reaction with a Cys-containing 16-mer peptide (PEP: AVMNNVTCTRIYEKVE. The sequence is derived from the redox active C127 in FABP5 with neighboring amino acids) (Fig. 2B). PEP reaction with dhG resulted in a single product peak in the HPLC chromatogram (Fig. 2B) that corresponds to the Michael reaction conjugation confirmed by mass spectrometry (Fig. S1C, ESI†), suggesting the selective reaction of dhG with Cys. The reactions of Michael acceptors, such as an acryl group, with thiols proceed at high rates (the second-order rate constant 0.25–65.0 M^−1^ s^−1^).^34^ Therefore, we monitored the reaction kinetics of dhG with fluorescein-conjugated PEP (FAM-PEP). To measure the reaction rate, FAM-PEP conjugation with dhG (over 10-fold excess, pH 8.0) was monitored over time in the urea-based gel electrophoresis (Fig. 2C).^35^ FAM-PEP showed time- and dose-dependent dhG conjugation (Fig. S1D, ESI†) with the second-order rate constant of 53.6 M^−1^ min^−1^ (Fig. 2D). The kinetic analysis indicates that the half-life (*t*_1/2_) of FAM-PEP is 12.9 min (with 1 mM dhG), suggesting 90% conversion in <1 h. These experiments confirm that dhG selectively reacts with Cys, leading to a thioether-based glutathione modification on Cys.

### dhG induces a glutathione modification at Cys127 of FABP5

**Fig. 3:**
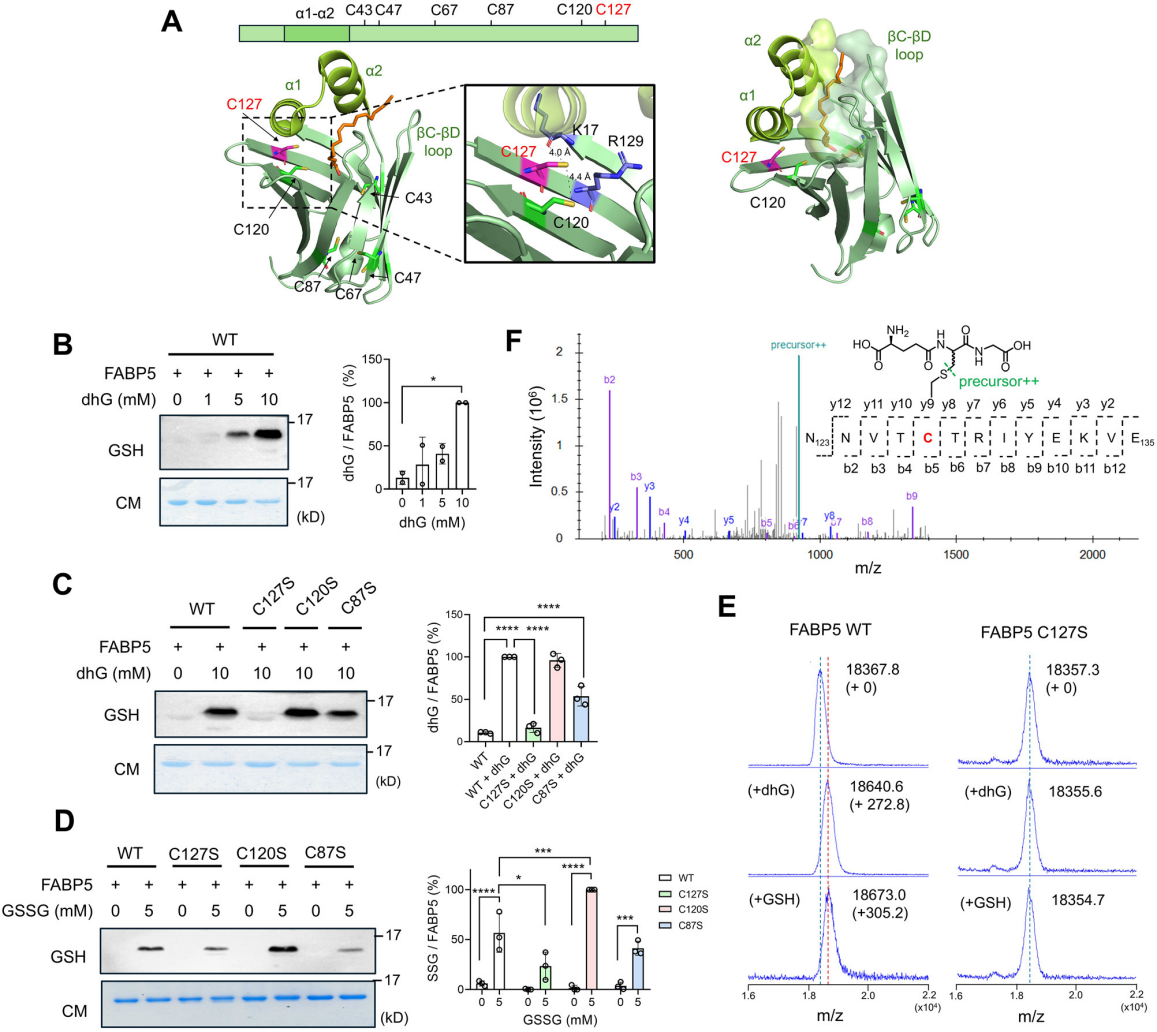
dhG induces glutathione modification on C127 of FABP5. (A) FABP5 structure (PDB: 4LKT) with positions of 6 cysteine residues. FABP5 has a twisted β-barrel structure with two helices (α1 and α2) acting as a lid (left). An enlarged structure around C127 with residues in proximity (middle). The size and depth of the lipid-binding pocket in FABP5 (right). Linoleic acid is shown in a stick model (orange). (B) dhG-modification on FABP5 WT. Increasing amounts of dhG were incubated with purified FABP5 in PBS, which was analyzed by Coomassie stain (CM) and glutathione antibody (GSH) (*n* = 2, biological replicates). (C) dhG-modification on FABP5 WT and cysteine mutants (*n* = 3, biological replicates). (D) GSSG-mediated *S*-glutathionylation of FABP5 WT and cysteine mutants. Purified FABP5 constructs were incubated with GSSG for 1 h (*n* = 3, biological replicates). (E) MALDI-TOF analysis of FABP5 WT or C127S incubated with dhG or GSSG. FABP5 constructs were incubated with dhG (10 mM) or GSSG (5 mM) for 1 h (*n* = 3, biological replicates). (F) MS2 spectrum of a dhG-modified peptide in FABP5. FABP5 modified by dhG was digested by CNBr and analyzed by LC-MS/MS, finding a peptide modified by dhG at C127. Data show the mean ± SD (B)–(D) and are representative of replicate experiments (B)–(F). The statistical difference was analyzed by one-way (B) and (C) or two-way (D) ANOVA with Tukey's *post hoc* test, where *p < 0.03, **p < 0.002, ***p < 0.0002, ****p < 0.0001.

To evaluate dhG with protein, we selected FABP5, known for its cysteine oxidation. FABP5 has six cysteines (Fig. 3A), among which C120 and C127 are in proximity (4.4 Å) and susceptible to disulfide bond formation,^30,32^ and C127 is reported to form *S*-sulfenylation^36^ and *S*-glutathionylation,^17,31^ suggesting C127 as a redox-active cysteine.

We expressed and purified FABP5 from *E. coli*, and bound lipids were removed by delipidation (Fig. S2, ESI†). The dhG incubation with FABP5 caused dhG concentration-dependent modification detectable by glutathione antibody (Fig. 3B). dhG modification in FABP5 was not reduced upon DTT treatment, whereas the same DTT treatment reduced the level of *S*-glutathionylation in FABP5 induced by oxidized glutathione (GSSG) (Fig. S3A, ESI†), confirming the non-reducible nature of dhG modification in FABP5.

To analyze modified cysteines, FABP5 WT and individual cysteine mutants were compared. dhG induced glutathione modification in C120S and C87S with comparable or slightly reduced levels to WT. In contrast, dhG modification in C127S was the most significantly reduced compared to WT (Fig. 3C), suggesting that C127 is the major cysteine for dhG modification. Next, dhG modification was compared with *S*-glutathionylation. FABP5 constructs incubated with GSSG showed a similar pattern to dhG, such that FABP5 C127S showed the weakest signal of *S*-glutathionylation compared to WT, C120S, and C87S (Fig. 3D). Interestingly, FABP5 C120S shows higher *S*-glutathionylation than WT, which is likely attributed to the fact that WT can form an intramolecular disulfide bond between C127 and C120, reducing the level of *S*-glutathionylation.

To confirm dhG-modified cysteine sites, FABP5 constructs without and with dhG incubation were analyzed by MALDI-TOF. The analysis showed that dhG induced +273 Da addition to FABP5 WT (18 641 *vs.* 18 368 Da with and without dhG. Expected mass change: +273.10 Da) (Fig. 3E, left middle), whereas no significant change was observed with C127S (18 356 *vs.* 18 357 Da with and without dhG) (Fig. 3E, right middle). In comparison, the incubation of GSSG caused +305 Da addition to FABP5 WT (18 673 *vs.* 18 368 Da with and without GSSG. Expected mass change: +305.2 Da) (Fig. 3E, left, bottom) without observing a significant mass change with FABP5 C127S (18 355 *vs.* 18 357 Da with and without GSSG) (Fig. 3E, right, bottom). Subsequently, FABP5 WT modified by dhG was digested by cyanogen bromide (CNBr). The MALDI-TOF analysis of the fragmented mixture identified the mass matching to a peptide modified with dhG at C127 *via* Michael addition (NNVTC*TRIYEKVE, *m/z* 1842 Da) (Fig. S3B, ESI†). The subsequent LC-MS/MS analysis confirmed dhG modification at C127 (Fig. 3F). These experiments support that FABP5 C127 is the most susceptible to dhG modification *via* the Michael reaction, generating a thioether form of non-reducible glutathione modification in FABP5. dhG modification occurs on the same cysteines as *S*-glutathionylation in FABP5.

### FABP5 dhG modification increases its binding affinity to fatty acid

FABP5 has a twisted β-barrel structure, composed of two β-sheets arranged by ten β-strands, and two helices (α1 and α2) acting as a lid on top of the β-barrel (Fig. 3A).^32^ A fatty acid binds to the inner space of the β-barrel with U- or L-shape conformation (Fig. 3A, right),^37^ by which the helical lid moves in or out from the β-barrel core, increasing or decreasing α2 helix interaction with a βC-βD loop (*i.e.*, M35 and L60).^37^ C127 is relatively hidden in the β-barrel core, albeit close to the helix lid (Fig. 3A). Interestingly, FABP5 C127 *S*-glutathionylation was shown to increase its binding affinity to linoleic acid (LA) in a biochemical pull-down experiment.^31^ To demonstrate the functional similarity of FABP5 dhG modification to *S*-glutathionylation, we examined the binding affinity of FABP5 with LA after dhG modification or *S*-glutathionylation.

**Fig. 4:**
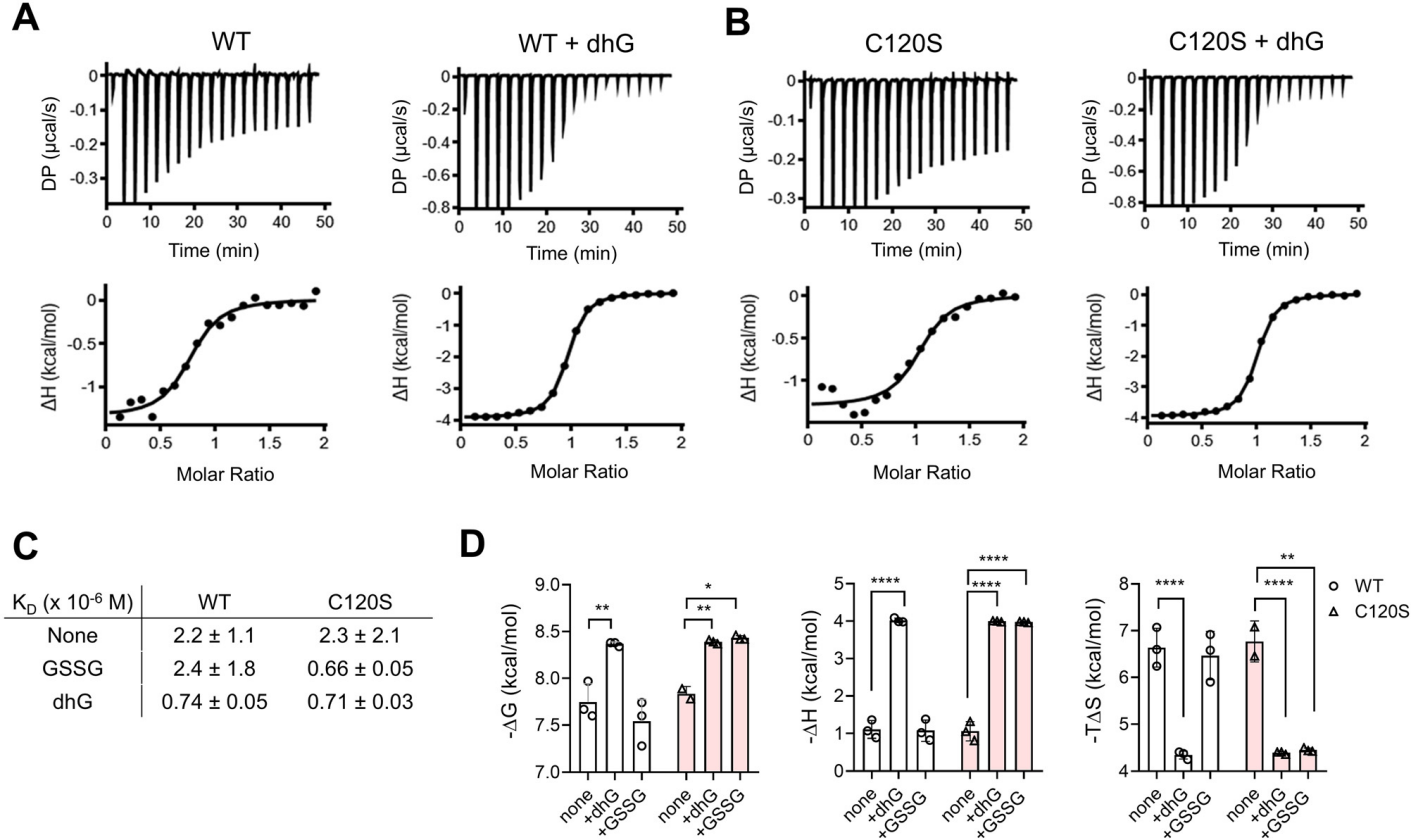
dhG-modification of FABP5 increases the binding affinity with linoleic acid. (A) and (B) The isothermal titration calorimetry (ITC) to measure FABP5 binding affinity with linoleic acid (LA) upon dhG modification. FABP5 WT (A) and C120S (B) without or with incubation of dhG (10 mM) were purified before the measurement by ITC (*n* = 3, biological replicates). The differential power (DP) was measured while LA (1 mM) was added to FABP5 (0.1 mM) in Tris–HCl, pH 7.4, over time. (C) The summary of the binding affinity between LA and FABP5 constructs without or with modification by dhG or GSSG. (D) The thermodynamic parameters of FABP5 binding interactions to LA. Data show the mean ± SD (C) and (D) and are representative of replicate experiments (A) and (B). The statistical difference was analyzed by two-way ANOVA with Tukey's *post-hoc* test (D), where *p < 0.03, **p < 0.002, ***p < 0.0002, ****p < 0.0001.

The isothermal titration calorimetry (ITC) experiment demonstrated that FABP5 WT binds to LA with a *K*_D_ value of 2.2 ± 1.1 μM (Fig. 4A, left, and Fig. 4C). In contrast, after dhG modification, FABP5 WT displayed *ca.* 3-fold higher binding affinity (*K*_D_ = 0.74 ± 0.05 μM) (Fig. 4A, right, and Fig. 4C), consistent with the observation that FABP5 *S*-glutathionylation increases its binding with LA.^31^ However, after GSSG incubation, FABP5 WT displayed binding affinity (*K*_D_ = 2.4 ± 1.8 μM) similar to non-glutathionylated FABP5 WT (Fig. 4C and Fig. S4, ESI†). This discrepancy was thought to occur because FABP5 WT *S*-glutathionylation at C127 may have caused C120 to displace the glutathione on C127, forming an intramolecular disulfide during the purification steps. Indeed, FABP5 WT incubated with GSSG showed a loss of *S*-glutathionylation signal over time, supporting the reversibility of C127 *S*-glutathionylation *in vitro* during the purification steps (Fig. S3C, ESI†). To remove the complication resulting from C120/C127 disulfide, we analyzed FABP5 C120S. FABP5 C120S retained similar binding affinity (*K*_D_ = 2.3 ± 2.1 μM) (Fig. 4B, left, and Fig. 4C) comparable to FABP5 WT, suggesting that C120S mutation does not cause a significant change in its binding to LA. FABP5 C120S increased its binding affinity to LA after dhG modification (*K*_D_ = 0.71 ± 0.03 μM) (Fig. 4B, right, and Fig. 4C) or GSSG incubation (*K*_D_ = 0.66 ± 0.05 μM) (Fig. 4C and Fig. S4, ESI†). The increased binding energy (Δ*G* = −8.4 *vs.* −7.7) of FABP5 WT with LA upon dhG modification is driven by more favorable enthalpy (Δ*H* = −4.0 *vs.* −1.0) and less unfavorable entropy (Δ*S* = −4.3 *vs.* −6.6) (Fig. 4D, bars 1 *vs.* 2). FABP5 C120S showed essentially the same thermodynamic changes as WT upon dhG modification (Fig. 4D, bars 4 *vs.* 5). In addition, GSSG-induced *S*-glutathionylation caused the same thermodynamic changes as dhG modification in FABP5 C120S (Fig. 4D, bars 6 *vs.* 5). These experiments support that FABP5 C127 glutathione modification increases the binding affinity to LA, and dhG-induced FABP5 glutathione modification exhibits a comparable functional effect to FABP5 *S*-glutathionylation.

### FABP5 glutathione modification increases its nuclear level and PPARβ/δ activation

The second step of our G-PROV strategy is to deliver the dhG-modified POI to cells (Fig. 1). To do so, we used a fusogenic liposome, which has been demonstrated to deliver cargo protein to the cytoplasm *via* fusion with the plasma membrane, as opposed to the lysosome or endosome *via* endocytosis.^38^ First, we analyzed the delivery of FABP5 to MCF7 cells, which have a low level of endogenous FABP5.^39^ The fusogenic liposome containing FLAG-FABP5 was incubated with MCF7 cells (1 h). The immunostaining of FLAG-FABP5 was found to be largely distributed in the cytoplasm without localizing to the nucleus, while the FLAG-signal was minimally overlapped with the endosome marker Rab9A (Fig. S5, ESI†),^40^ supporting that FABP5 was delivered mostly to the cytoplasm, but with a low level at the endosome/lysosome.

**Fig. 5:**
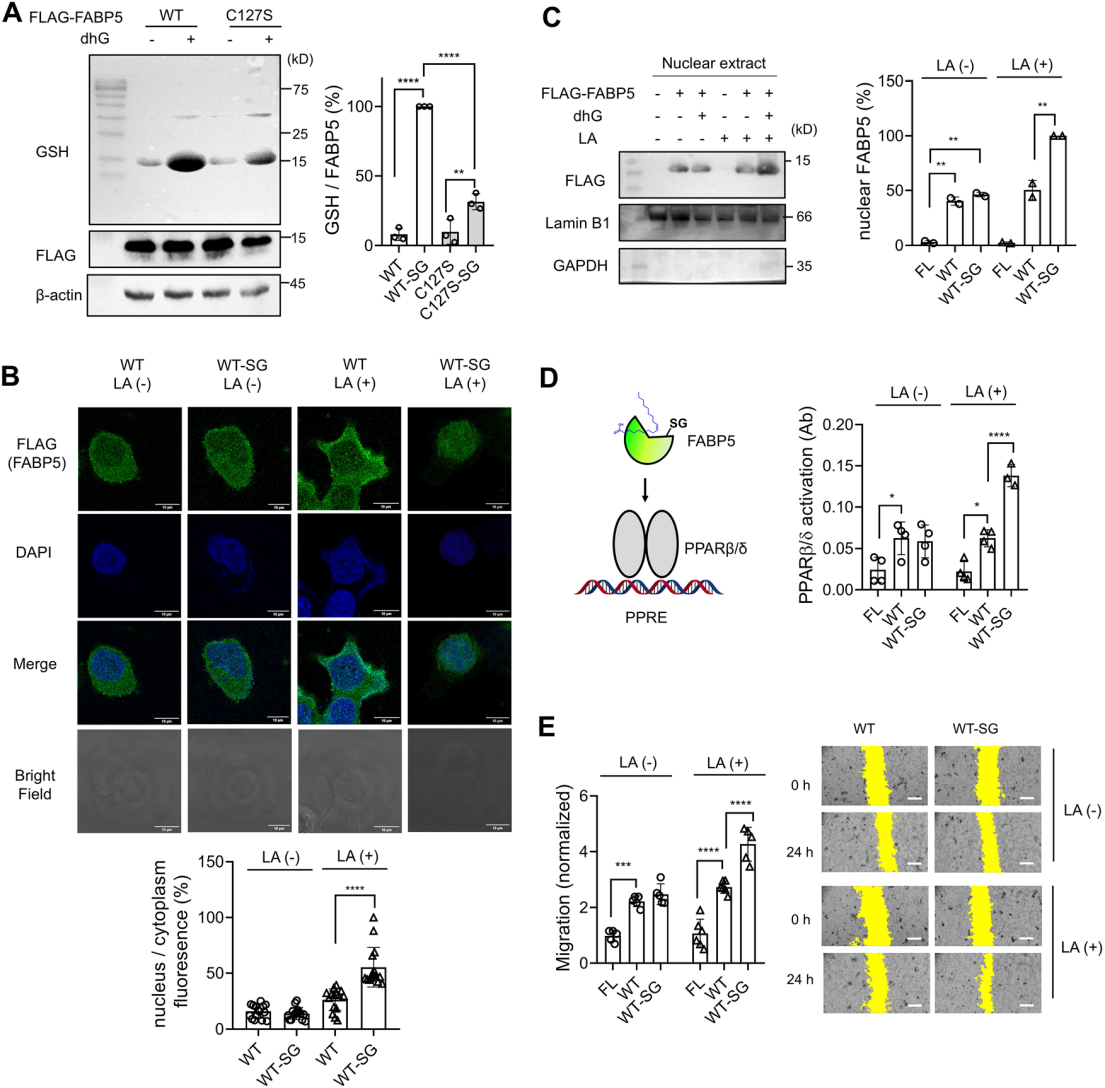
FABP5 glutathione modification increases its nuclear translocation, PPARβ/δ activation, and MCF7 cell migration in response to linoleic acid. Fusogenic liposomes alone (FL) or containing FABP5 WT or C127S without or with dhG modification (*i.e.*, WT, C127S, WT-SG, or C127S-SG) were incubated in MCF7 cells for 1 h. (A) Analysis of FABP5 in MCF7 cells after incubating fusogenic liposomes. Lysates were analyzed by western blots (*n* = 3, biological replicates). (B) Localization of FLAG-FABP5 upon adding linoleic acid (LA). After incubation of LA for 1 h, FABP5 localization (FLAG, green) was analyzed by immunostaining along with DAPI (blue) (*n* = 10 images out of 2 biological replicates). A scale bar = 10 μm. (C) FABP5 nuclear level upon adding LA. MCF7 cells were treated with none or LA for 12 h. MCF7 cells were lysed, and nuclear extracts were analyzed by western blots (*n* = 2, biological replicates). (D) PPARβ/δ activation upon adding LA. After incubating LA for 12 h, the nuclear extracts were collected, and the levels of PPARβ/δ bound to the peroxisome proliferator response element (PPRE) were measured by absorbance (*n* = 4, biological replicates). (D) The *in vitro* scratch migration assays of MCF7 cells upon incubating LA for 24 h. After incubating fusogenic liposomes containing FABP5 WT, MCF7 cells were incubated without or with LA. The images were taken at 0 and 24 h (*n* = 5 images out of 3 biological replicates). Yellow colors indicate the area without cells. A scale bar = 0.5 mm. Data show the mean ± SD (A)–(E) and are representative of replicate experiments (A)–(C) and (E). The statistical difference was analyzed by one-way (A) and (B) or two-way (C)–(E) ANOVA and Tukey's *post-hoc* test, where *p < 0.03, **p < 0.002, ***p < 0.0002, ****p < 0.0001.

The cellular delivery of FLAG-FABP5 constructs *via* fusogenic liposomes was also analyzed in lysates. The western blot analysis by FLAG-antibody found that the same amounts of four constructs (FABP5 WT and C127S without and with dhG) were delivered to cells (Fig. 5A, FLAG). In contrast, glutathione modification is mainly found in cells with FABP5 WT incubated with dhG (FABP5 WT-SG), along with a low level in FABP5 C127S incubated with dhG (FABP5 C127S-SG) (Fig. 5A, lanes 2 *vs.* 1, 3, 4). Notably, one distinct protein band, corresponding to FABP5 molecular weight, shows a strong signal for glutathione modification (Fig. 5A, lane 2), suggesting that mainly a single protein, FABP5, retains a significant level of glutathione modification in the whole proteome.

FABP5 upon binding to LA generates its non-canonical nuclear localization signal.^37^ FABP5 was previously shown to increase its translocation to the nucleus in response to H_2_O_2_,^31^ implying that FABP5 *S*-glutathionylation increases its binding to LA and enhances its nuclear localization. The translocated FABP5 interacts with PPARβ/δ for transcriptional activation.^41^ Therefore, the nuclear translocation of FABP5 constructs in response to LA was examined. Without adding LA, FABP5 WT was largely seen in the cytoplasm with a low level in the nucleus (Fig. 5B, column 1, and Fig. S6A (ESI†); the percentage of FABP5 in the nucleus over the cytoplasm, *P*_*n*/*c*_ = 15.6 ± 5.9%, *n* = 10). The incubation of LA slightly increased the level of FABP5 WT in the nucleus but without statistical significance (Fig. 5B, column 3, and Fig. S6C (ESI†); *P*_*n*/*c*_ = 26.1 ± 9.7%, *n* = 10). Similarly, FABP5-SG was also found at a low level in the nucleus without LA (Fig. 5B, column 2, and Fig. S6B (ESI†); *P*_*n*/*c*_ = 13.9 ± 5.2%, *n* = 10). However, FABP5-SG was significantly found in the nucleus upon adding LA (Fig. 5B, column 4, and Fig. S6D (ESI†); *P*_*n*/*c*_ = 55.3 ± 17.7%, *n* = 10), displaying increased translocation of FABP5-SG over FABP5 in response to LA. In addition, the nuclear levels of four FABP5 constructs in response to LA were examined by detecting FABP5 in the nuclear lysates after cell fractionation. FABP5 WT-SG did not show its increased nuclear level, compared to FABP5 WT, without adding LA (Fig. 5C, lane 3 *vs.* 2). However, FABP5 WT-SG was more significantly found in the nuclear extract than FABP5 WT upon adding LA (Fig. 5C, lane 6 *vs.* 5), suggesting the enhanced translocation of FABP5 upon dhG modification. Lastly, the PPARβ/δ transcriptional activation was examined. The PPARβ/δ activation assay showed that FABP5 WT and FABP5 WT-SG activate PPARβ/δ at comparable levels without adding LA (Fig. 5D, bars 3 *vs.* 2). However, in the presence of LA, FABP5 WT-SG induced higher PPARβ/δ activation than FABP5 WT (Fig. 5D, bars 6 *vs.* 5). These experiments support that dhG-modified FABP5 glutathionylation increases nuclear translocation and PPARβ/δ activation.

### FABP5 glutathione modification increases cell migration

The PPARβ/δ activation induces higher migration, invasion, and metastasis of cancer cells.^29,42,43^ Therefore, we analyzed the migration of MCF7 cells containing FABP5 constructs upon adding LA. The *in vitro* scratch migration assay showed that MCF7 cells containing FABP5 WT or FABP5 WT-SG showed comparable levels of cell migration in the absence of LA (Fig. 5E, bars 3 *vs.* 2). However, upon incubating LA, FABP5 WT-SG induced higher cell migration than FABP5 WT (Fig. 5E, bars 6 *vs.* 5). To see the importance of C127 for glutathione modification, we analyzed FABP5 C127S without and with glutathione modification by dhG. Unlike FABP5 WT, FABP5 C127S and FABP5 C127S-SG induced similar levels of MCF7 cell migration in the presence and absence of LA (Fig. S7, lanes 2 *vs*. 3, and 5 *vs.* 6, ESI†), supporting that FABP5 C127 glutathione modification is responsible for the observed increase in cell migration.

## Discussion

*S*-Glutathionylation is emerging as an important redox-regulatory event in pathophysiologic processes.^17^ Although a large number of *S*-glutathionylated proteins were identified *via* proteomics, the functional annotation of the identified *S*-glutathionylated cysteines remains limited.^17^ In this report, we developed a strategy named G-PROV that installs glutathione modification on a protein of interest (POI) with its subsequent delivery to cells, where the functional effects of “glutathione” modification in the POI can be probed. It is worth stating that the proteome in cells largely stays in a reduced state under basal (non-stressed) conditions. Therefore, the cellular delivery of the POI modified by dhG *via* the fusogenic liposome rendered cells where only the POI retains a significant glutathione modification in the proteome, and the effect of glutathionylation on a single protein can be investigated. Therefore, the G-PROV approach helps determine “phenotype” changes resulting from POI “glutathione” modification.

Previously, the “Tag-and-Modify” approach for converting cysteines in a recombinant protein to dehydroalanine (dhA) (*e.g.*, using diethyl *meso*-2,5-dibromo adipate) was developed.^44,45^ Subsequently, dhA in a protein could be further derivatized to diverse post-translational modifications (PTMs), including glutathionylation.^44^ Therefore, the “Tag-and-Modify”-mediated non-reducible glutathione modification in proteins is feasible,^44^ but the approach typically accompanies mutations of cysteines other than cysteines of interest. In addition, the two-step process may involve additional purification steps. As an alternative, the G-PROV strategy demonstrates a simple one-step procedure for glutathione modification in proteins, combined with a strategy for its cellular delivery for functional analysis. However, it is important to note that the Michael reaction between dhG and cysteine in the protein generates glutathione modification with a loss of stereochemistry in Cys of glutathione and one atom shorter than *S*-glutathionylation. In addition, as opposed to the dynamic and reversible nature of *S*-glutathionylation, the dhG-mediated glutathionylation is irreversible. Therefore, although we demonstrate that dhG-modification in FABP5 induces similar functional changes to *S*-glutathionylation, it is possible that dhG-mediated glutathionylation does not recapitulate biological phenotypes resulting from reversible changes of glutathionylation or cause biochemical changes deviating from physiological *S*-glutathionylation. Moreover, dhG reacts with any cysteines, where the selectivity is governed by their nucleophilicity and accessible surface area (ASA). Therefore, dhG could react with multiple cysteines in a POI, limiting site-specific functional analysis. In this case, it will be important to include mutant controls, such as C127S in FABP5, for functional analysis. Lastly, the G-PROV approach may pose a risk of delivering impurity proteins as well as the POI to cells, which can affect the biological phenotype. Thus, additional control experiments will be necessary to validate the observed phenotype.

FABP5 has six cysteines (C43, C47, C67, C87, C120, and C127). The six cysteines are partially conserved in the FABP family, and FABP5 is the only member in the FABP family with six cysteines. C120–C127 disulfide was found previously,^32^ and C67–C87 are in proximity without forming a disulfide.^32^ In addition to the intramolecular disulfide, C127 was found for *S*-sulfenylation^46^ and *S*-glutathionylation,^31^ suggesting its tendency to form multiple oxoforms with high nucleophilicity and oxidation susceptibility. In this report, we introduced a glutathione modification mainly at C127 in FABP5. The ITC experiment showed that dhG-modified or GSSG-mediated glutathionylation in FABP5 at C127 increases *ca.* 3-4-fold binding affinity. Interestingly, the increase in the binding affinity (more negative Δ*G*) is attributed to the enthalpy increase (more negative Δ*H*) in addition to the more favorable entropy (more positive Δ*S*), suggesting that glutathione could form additional interactions with LA directly or *via* a network of water molecules in a pocket. Thus, the data imply that the increased binding affinity of FABP5 may result from glutathione modification *per se* rather than from other oxoforms. However, it remains to be seen whether other oxoforms of C127 can increase the FABP5 binding affinity to LA. Lastly, we demonstrate that FABP5 glutathione modification increases MCF7 cell migration *via* activating PPARβ/δ. Because FABP5 is involved in activating many transcription factors and oncogenes (*e.g.*, NF-kB),^29^ it would be necessary to see whether FABP5 glutathionylation regulates other signaling pathways.

## Author contributions

Daniel Oppong: formal analysis, investigation, visualization, writing – original draft, writing – review & editing. Rayavarapu Padmavathi: formal analysis, investigation, visualization, writing – original draft, writing – review & editing. Dhanushika Kukulage: conceptualization, formal analysis, investigation, writing – review & editing. Madhu Shivamadhu: investigation, visualization, writing – review & editing. Elizabeth Newberry: formal analysis, writing – review & editing. Anneliese Faustino: formal analysis, investigation, visualization, writing – review & editing. Hsin-Yao Tang: supervision, writing – review & editing. Young-Hoon Ahn: conceptualization, formal analysis, supervision, visualization, writing – original draft, writing – review & editing, funding acquisition.

## Conflicts of interest

The authors declare that they have no conflicts of interest with the contents of this article.

## Supplementary Material

CB-006-D5CB00052A-s001

## Data Availability

The data supporting this article have been included as part of the ESI.† The mass spectrometry data have been deposited to the ProteomeXchange Consortium (https://proteomecentral.proteomexchange.org) *via* the PRIDE [1] partner repository with the dataset identifier PXD061319.
