# Supplementary material for: Dehydroglutathione, a glutathione derivative to introduce non-reversible glutathionylation
Source: RSC Chem Biol. 2025 May 21;6(7):1156–64. doi: 10.1039/d5cb00052a (PMC12143294; doi:10.1039/d5cb00052a)
Supplement: CB-006-D5CB00052A-s001 [file CB-006-D5CB00052A-s001.pdf]

Supplementary Information (SI) for RSC Chemical Biology.
This journal is © The Royal Society of Chemistry 2025

# Supplementary Information

# Dehydroglutathione, a glutathione derivative to introduce non-reversible glutathionylation

Oppong *et al*.

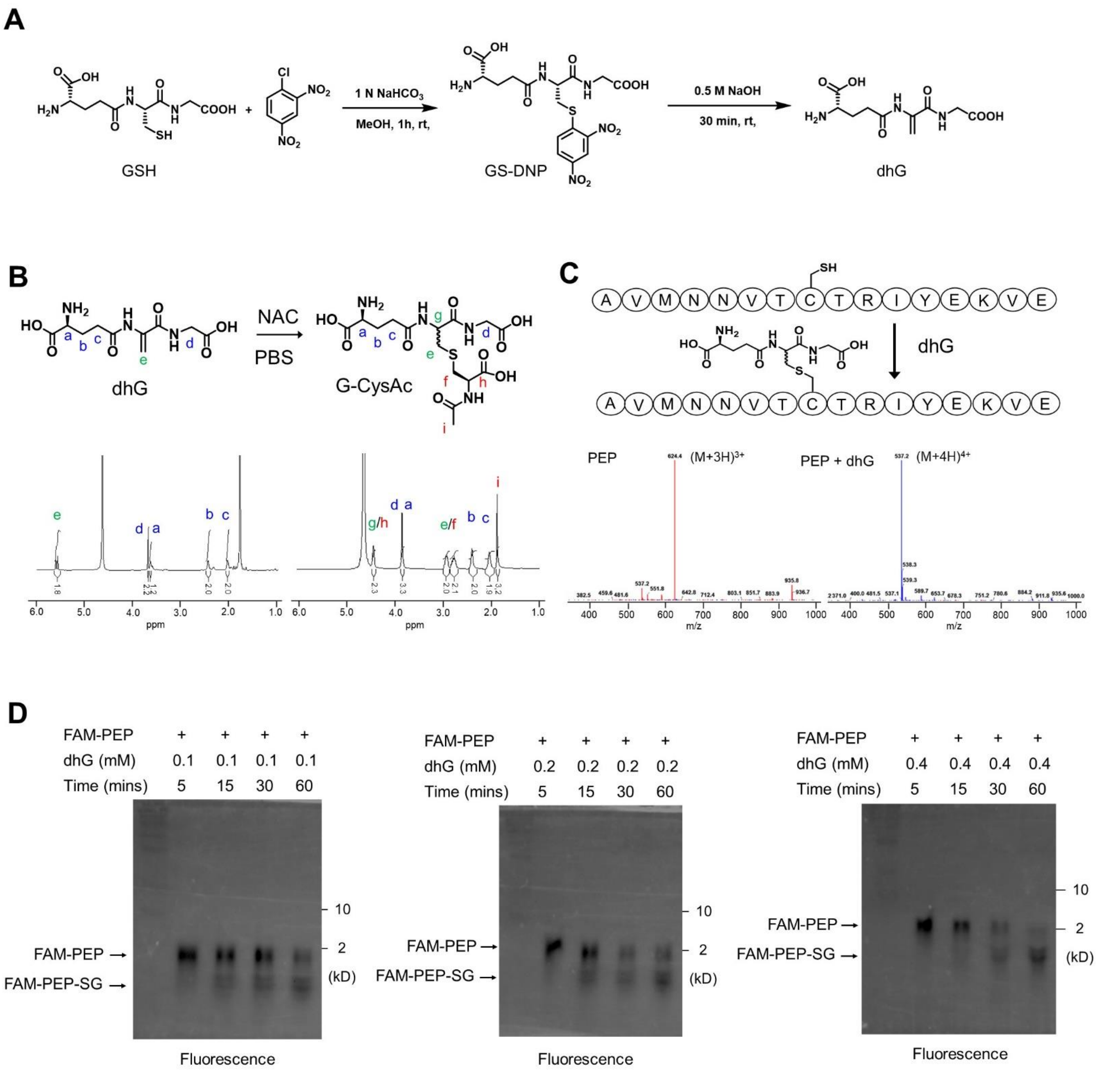


**Figure S1. dhG reaction with Cys.** (A) dhG synthesis. dhG was synthesized as reported.[S1] (B) dhG reaction with N-acetylcysteine (NAC). dhG and the reaction product were analyzed by NMR. (C) dhG reaction with Cys-containing peptide (PEP). PEP and its dhG-conjugation product were analyzed by HPLC (shown in Figure 2B) and ESI-MS. Calculated mass of PEP, 624.3 $(M+3H)^{3+}$; found mass, 624.3 $(M+3H)^{3+}$. Calculated mass of PEP-conjugated with dhG (PEP-SG), 536.5 $(M+4H)^{4+}$; found mass, 537.2 $(M+4H)^{4+}$. (D) dhG reaction with fluorescein-conjugated PEP (FAM-PEP). FAM-PEP was reacted with dhG in concentration- and time-dependent manners (n=3, biological replicate). Peptides were detected by fluorescence.

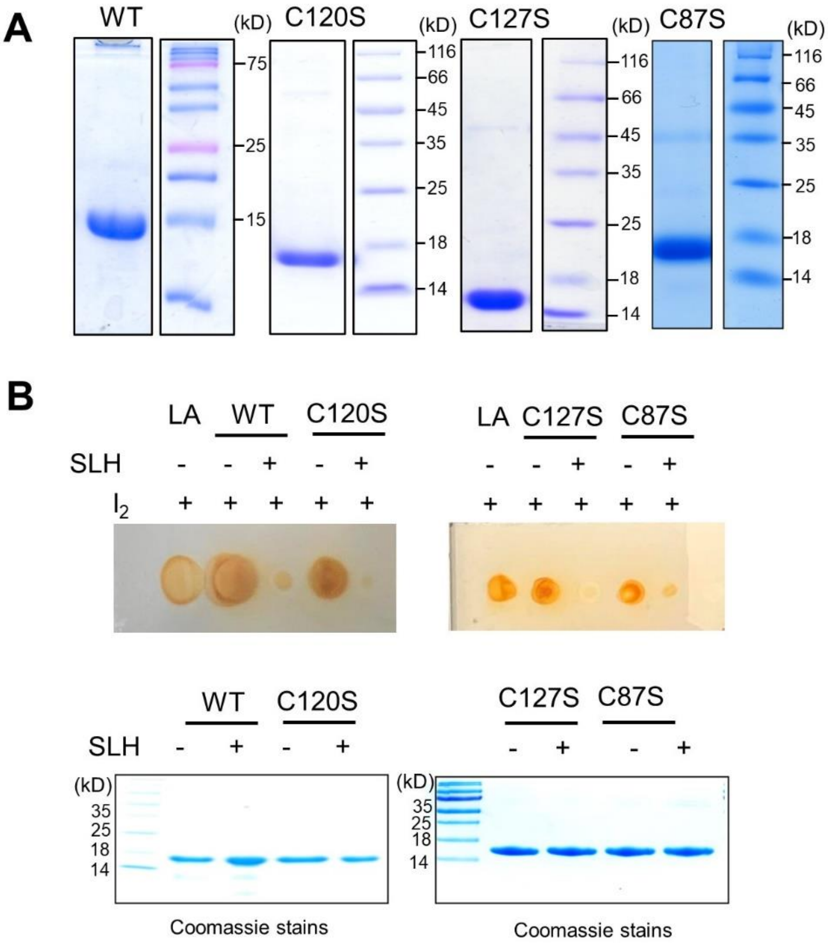


**Figure S2. Purification and delipidation of FABP5 constructs**. (A) The purity of FABP5 WT and mutants. Proteins were visualized by Coomassie stains. (B) Delipidation of FABP5. Purified FABP5 constructs were incubated with lipophilic Sephadex LH-20-100 (SLH) that bind and remove the bound fatty acids. The fatty acid contents in FABP5 constructs were monitored by the reaction with iodine ($I_2$). Linoleic acid (LA) was used as a positive control (lane 1) (top). FABP5 constructs incubated without and with Sephadex LH-20-100 were visualized by Coomassie stains (bottom).

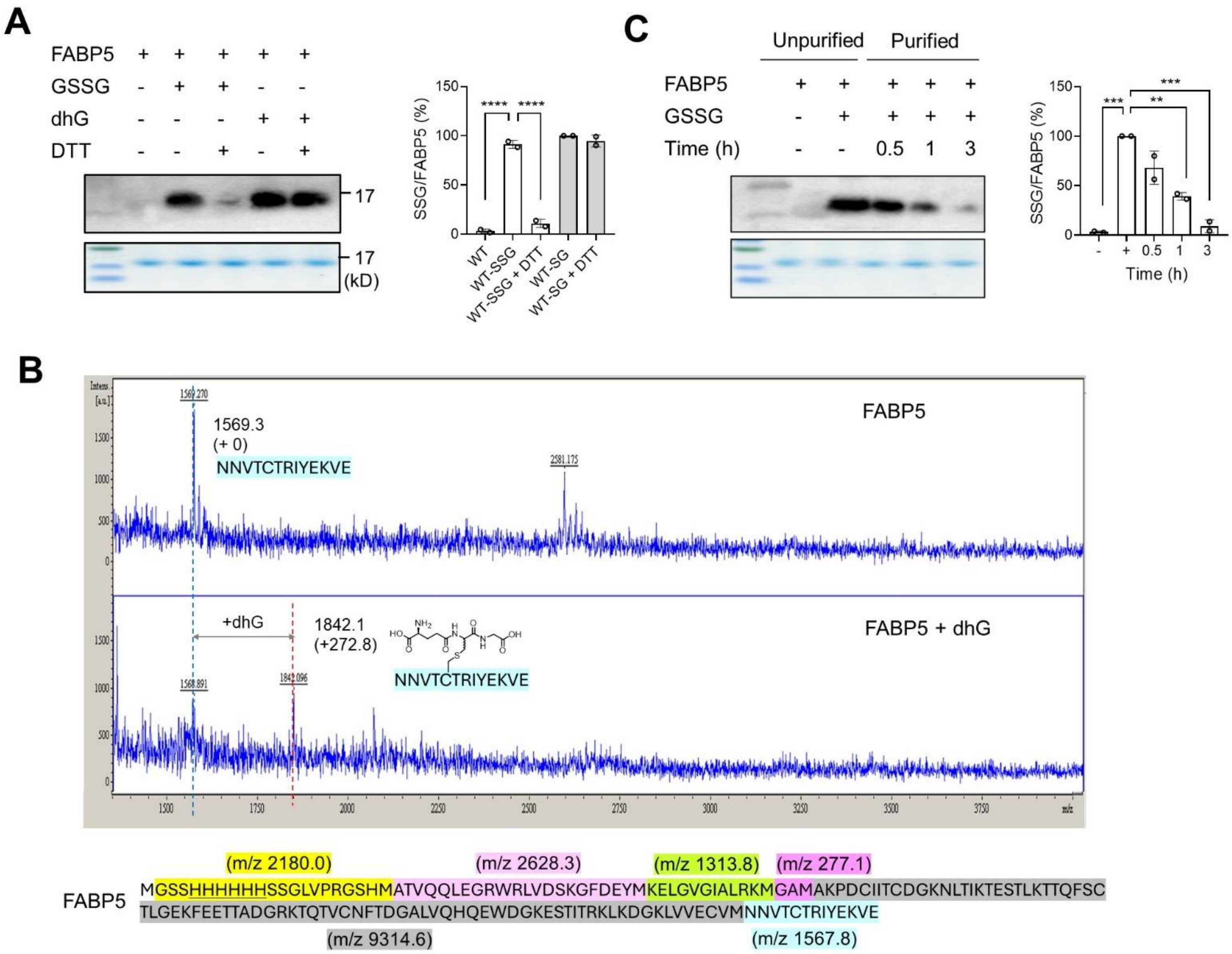


**Figure S3. In vitro analysis of FABP5 modification by dhG**. (A) dhG causes a non-reducible glutathione modification on FABP5. Purified FABP5 WT was incubated with dhG (10 mM) or GSSG (5 mM) in PBS in the absence or presence of DTT (20 mM) for 12 h. Glutathione modification was analyzed by western blot using glutathione antibody (n=2, biological replicate). (B) MALDI-TOF analysis of FABP5 modified by dhG after fragmentation by CNBr. FABP5, without or with dhG modification, was digested by CNBr overnight. The digests were analyzed by MALDI-TOF, monitoring the mass corresponding to the peptide containing C127 (NNVTCRIYEKVE) non-modified (top m/z 1569.3) or modified (bottom, m/z 1,842.1) by dhG. Recombinant His-tag FABP5 amino acid sequence is shown with different colors indicating peptides cleaved by CNBr. (C) FABP5 WT loses its glutathionylation upon purification. FABP5 WT was incubated in 1xPBS with GSSG (5 mM) for 12 h (unpurified). After GSSG incubation, FABP5 WT was purified by gel filtration (NAP-10 column). The collected FABP5 WT was left at room temperature and analyzed at different time points (purified) (n=2, biological replicate). Data show the mean ± SD (A, C) and are representative of replicate experiments (A-C). The statistical difference was analyzed by one-way ANOVA with Tukey's post-hoc test, where $^{*}p < 0.03$, $^{**}p < 0.002$, $^{***}p < 0.0002$, $^{****}p < 0.0001$.

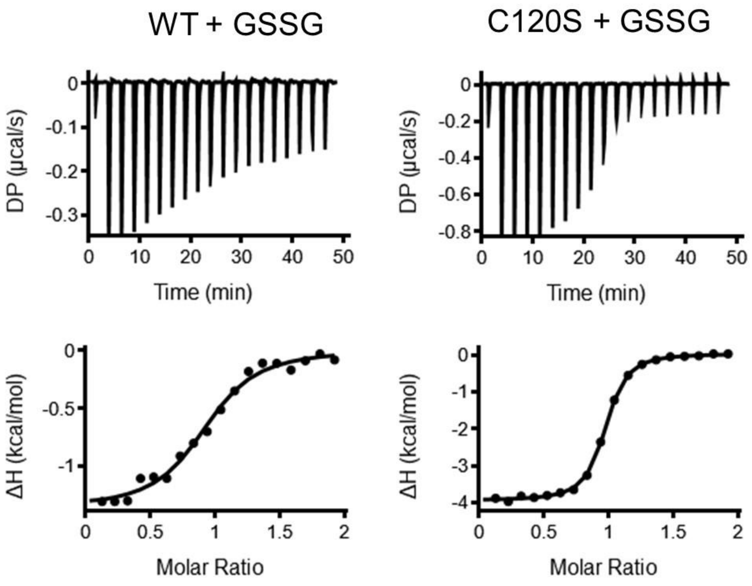


**Figure S4. FABP5 binding affinity to linoleic acid upon modification by GSSG.** FABP5 constructs were incubated with GSSG (5 mM) for 3 h and purified by gel filtration. The isothermal titration calorimetry (ITC) was used with FABP5 (0.1 mM) and linoleic acid (LA) (1 mM) in Tris-HCl, pH 7.4. The differential power (DP) was plotted as LA is added to FABP5 over time. Data show representatives of 3 biological replicate experiments.

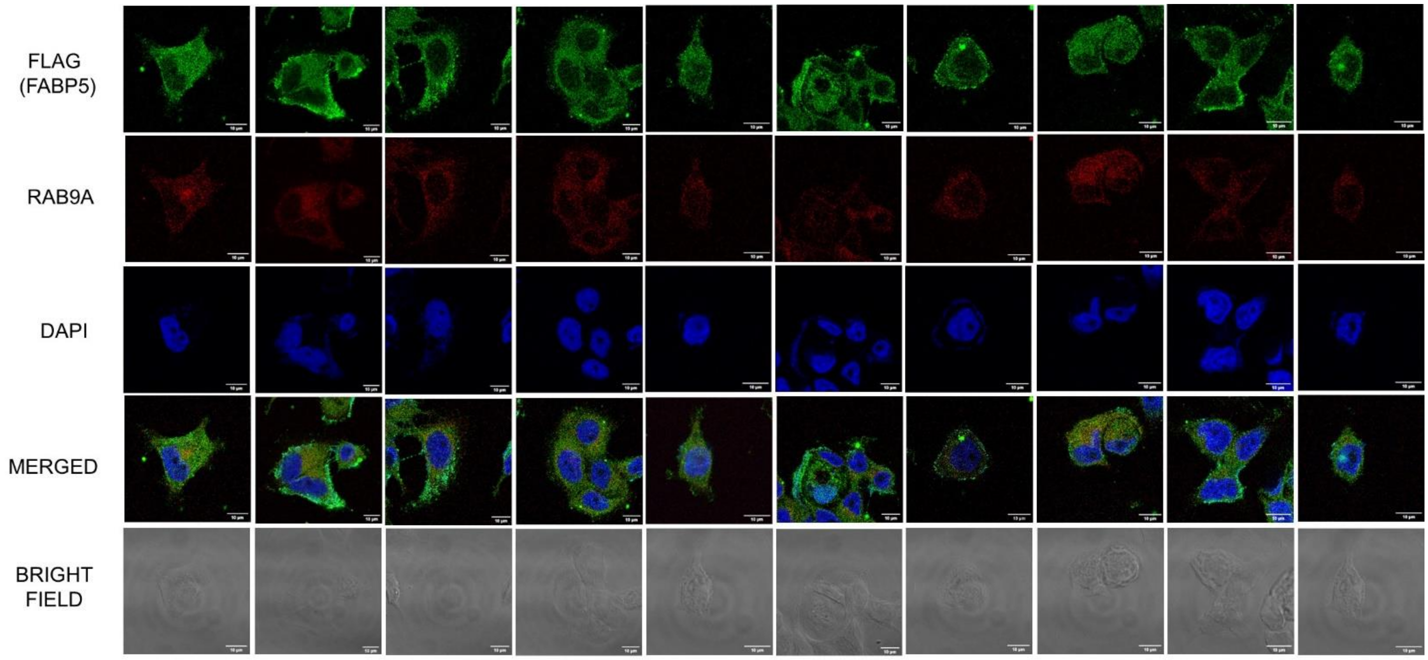


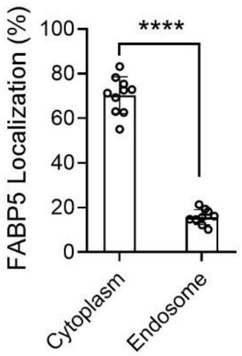


**Figure S5. Analysis of FABP5 in cells after being delivered by fusogenic liposome**. Localization of FLAG-FABP5 in confocal microscopy. MCF7 cells were incubated with fusogenic liposomes containing FABP5 constructs for 1 h. Cells were fixed and analyzed by immunostaining. Rab9A was used as an endosomal marker. The number of pixels displaying green only (FLAG) and both green/red (FLAG/Rab9A) were counted and presented as cytoplasm and endosome/lysosome, respectively (n=10 images out of 2 biological replicates). A scale bar = 10 µm. Data show the mean ± SD. The statistical difference was analyzed by two-tailed Student's unpaired t-test, where *$p < 0.03$, **$p < 0.002$, ***$p < 0.0002$, ****$p < 0.0001$.

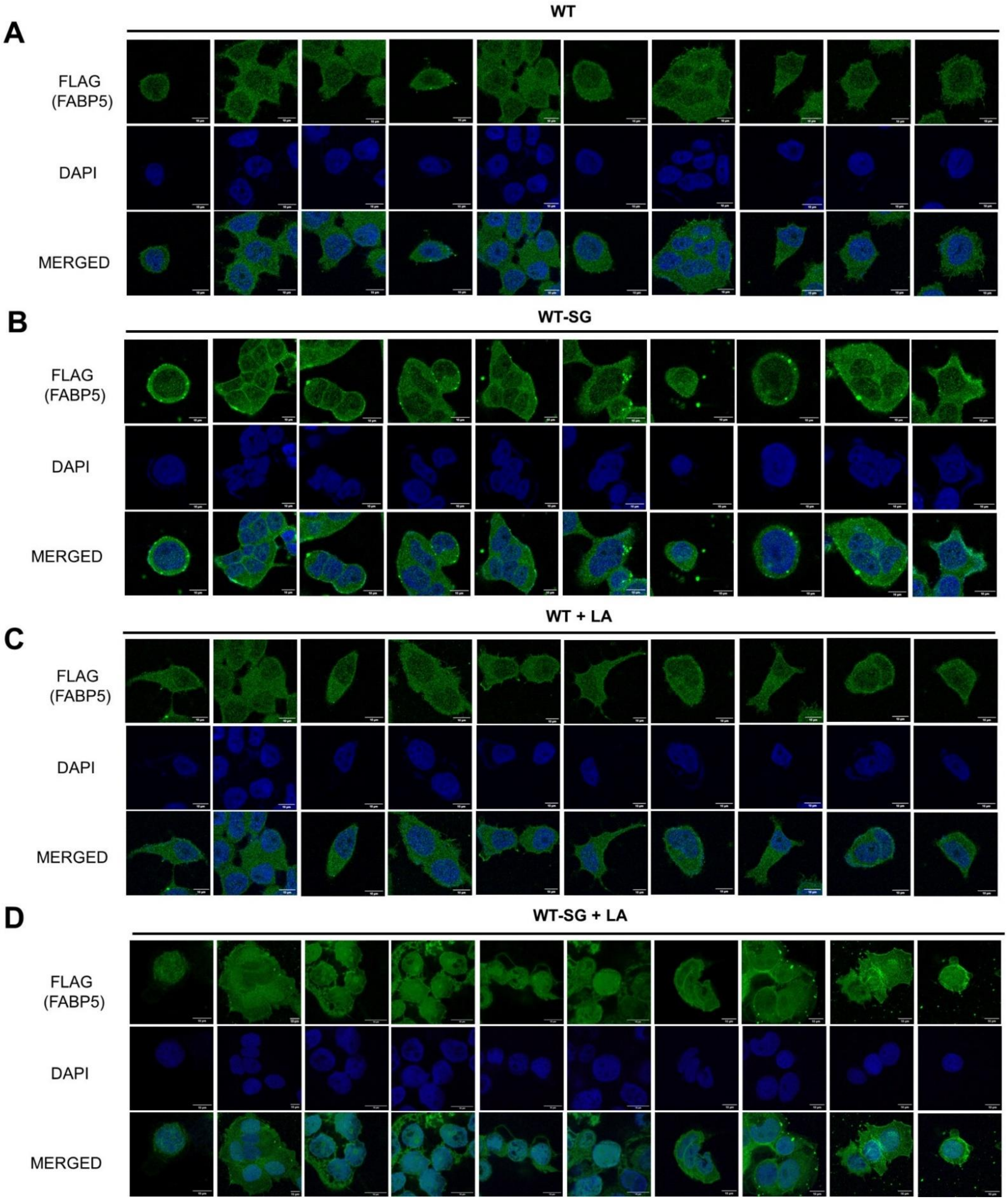


**Figure S6. Localization of FABP5 constructs upon adding LA**. (A-D) MCF7 cells were incubated with fusogenic liposomes containing FABP5 constructs for 1 h. Cells were fixed and analyzed by immunostaining with FLAG antibody (green, FABP5) and DAPI (blue). DAPI was used as a nucleus marker. DAPI was used to find the nucleus boundary. Green signals within and outside the nucleus boundary were quantified to indicate the localization of FABP5 in the nucleus and cytoplasm, respectively (n= 10 images out of 2 biological replicates). Replicates are shown, including those in Figure 5 B. A scale bar = 10 μm.

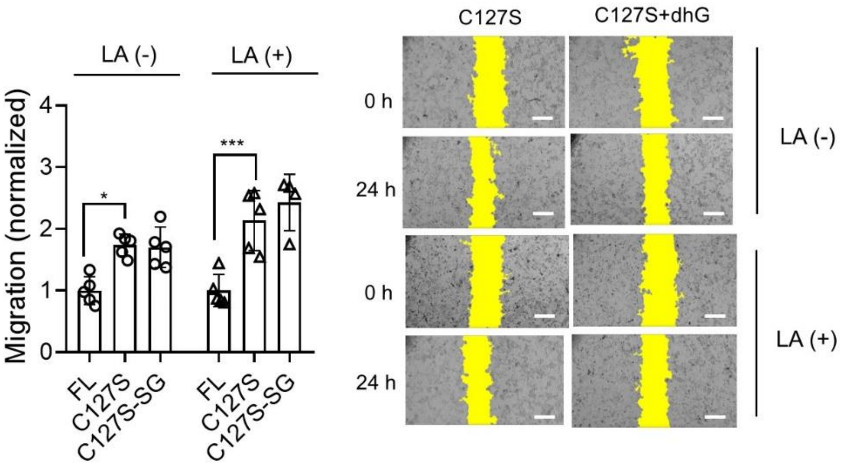


**Figure S7. The in vitro scratch migration assays of MCF7 cells containing FABP5 C127S.** In vitro scratch migration assay. After incubating fusogenic liposomes, cells were scratched and incubated with LA for 24 h (n=5 images out of 3 biological replicates). The yellow color indicates the area without cells. A scale bar = 0.5 mm. Data show the mean ± SD and representatives of replicate experiments. The statistical difference was analyzed by two-way ANOVA and Tukey's post-hoc test, where *p < 0.03, **p < 0.002, ***p < 0.0002, ****p < 0.0001.

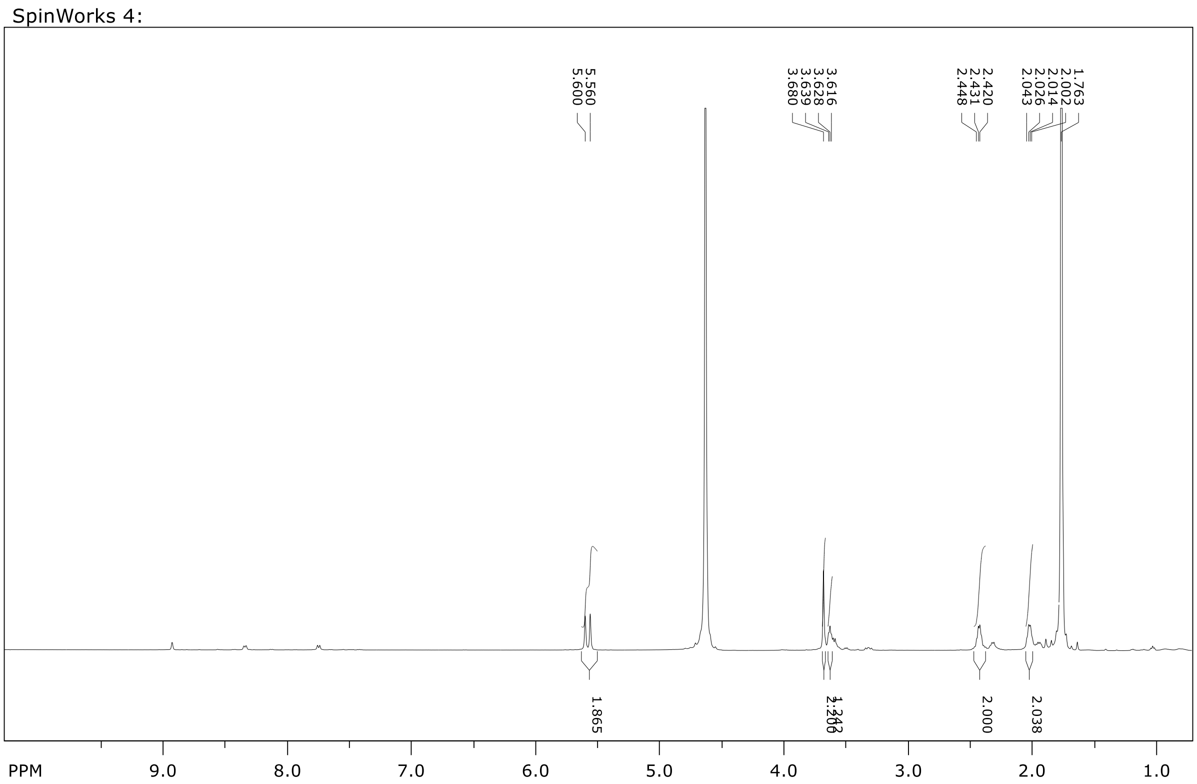


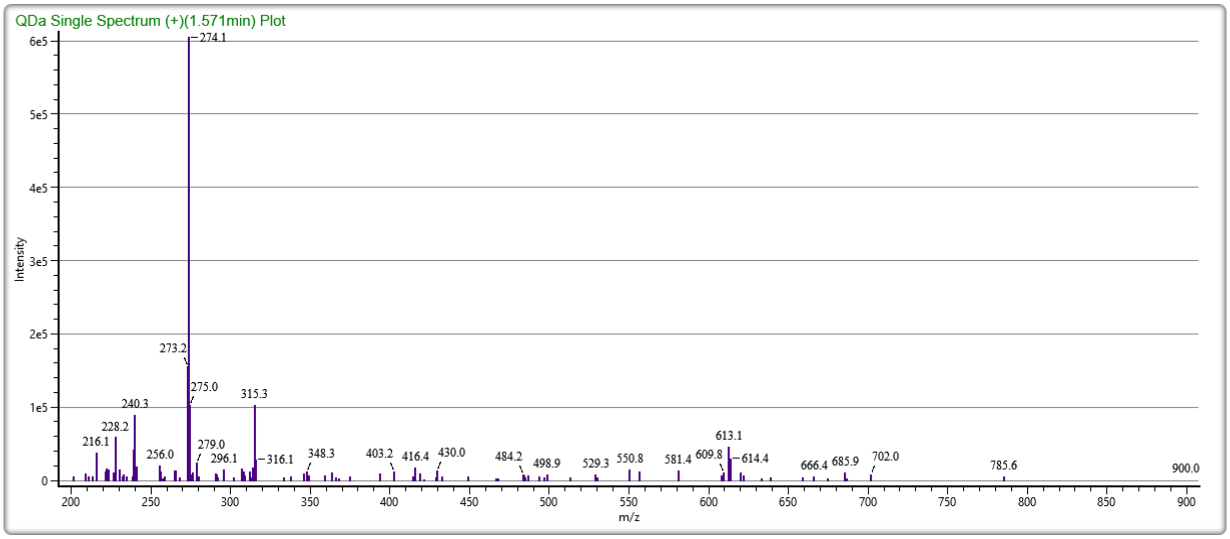


**Figure S8**. Characterization of dhG. $^1$H-NMR spectrum (top) and ESI-MS spectrum (bottom).

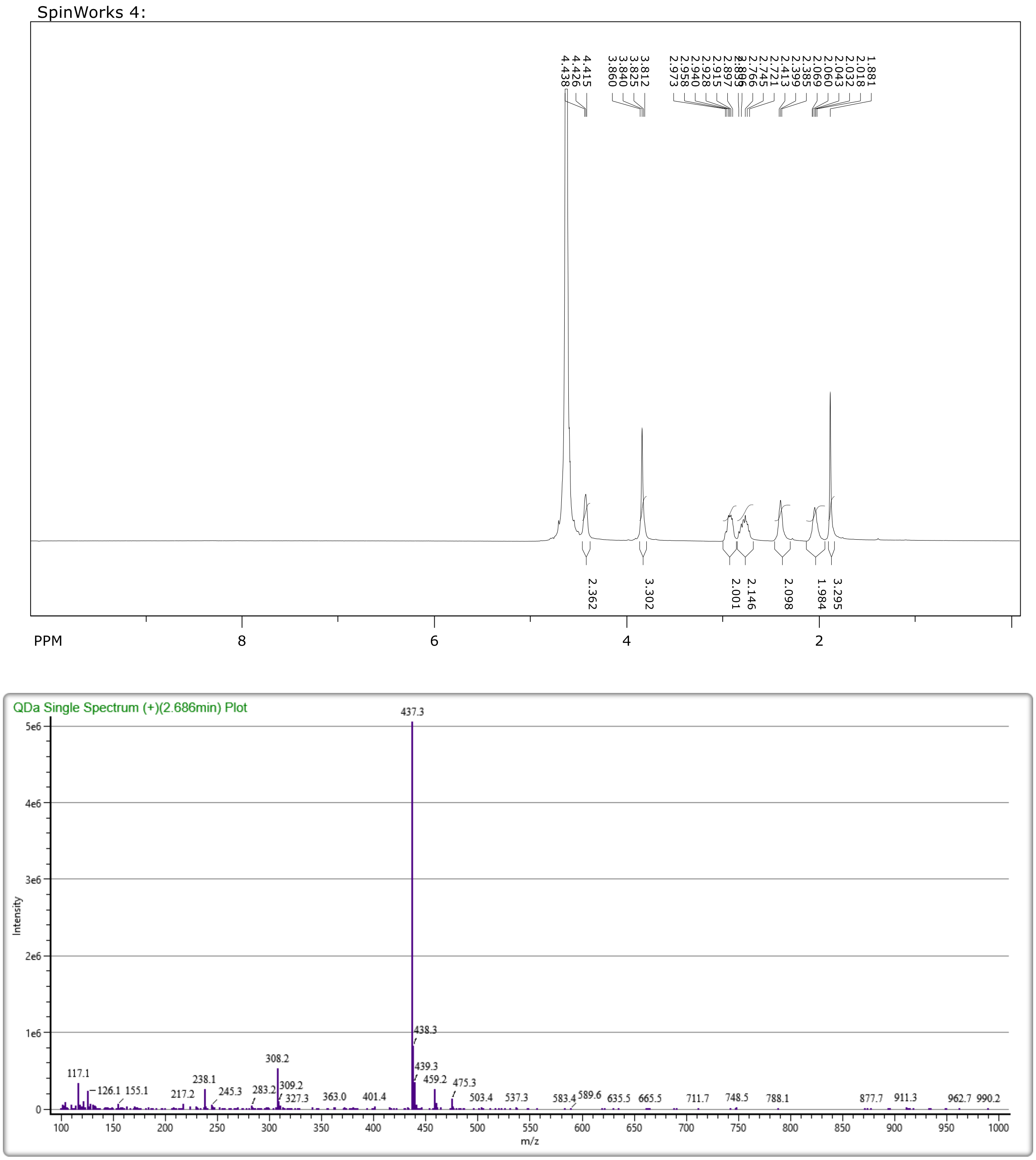


**Figure S9**. Characterization of dhG-conjugated N-acetyl-cysteine (G-Cys-NAC). $^1$H-NMR spectrum (top) and ESI-MS spectrum (bottom).

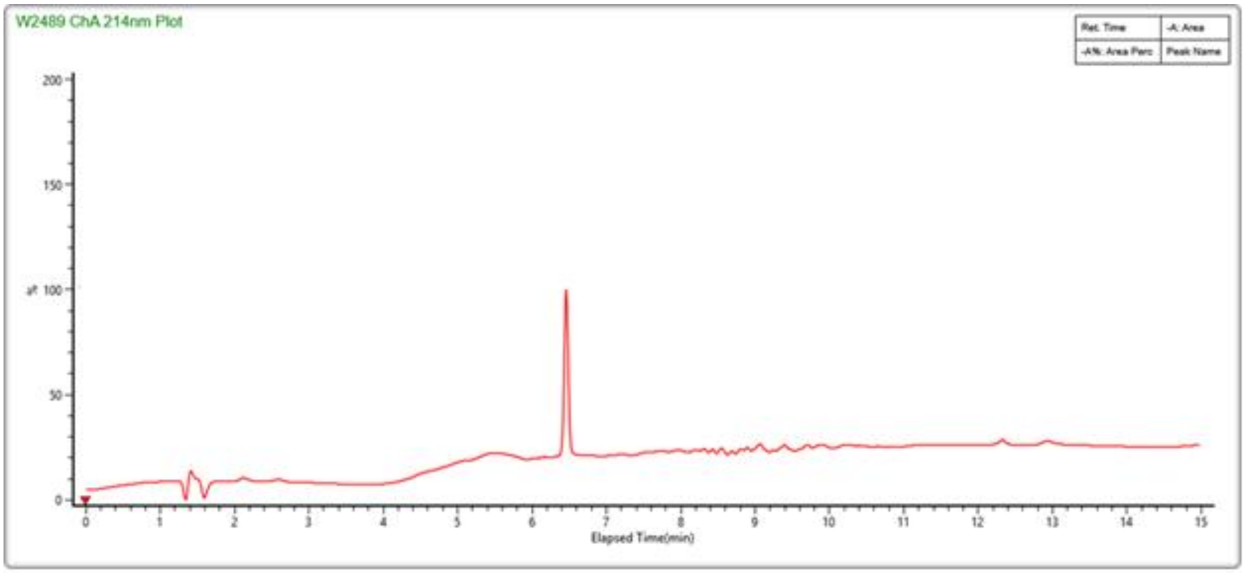


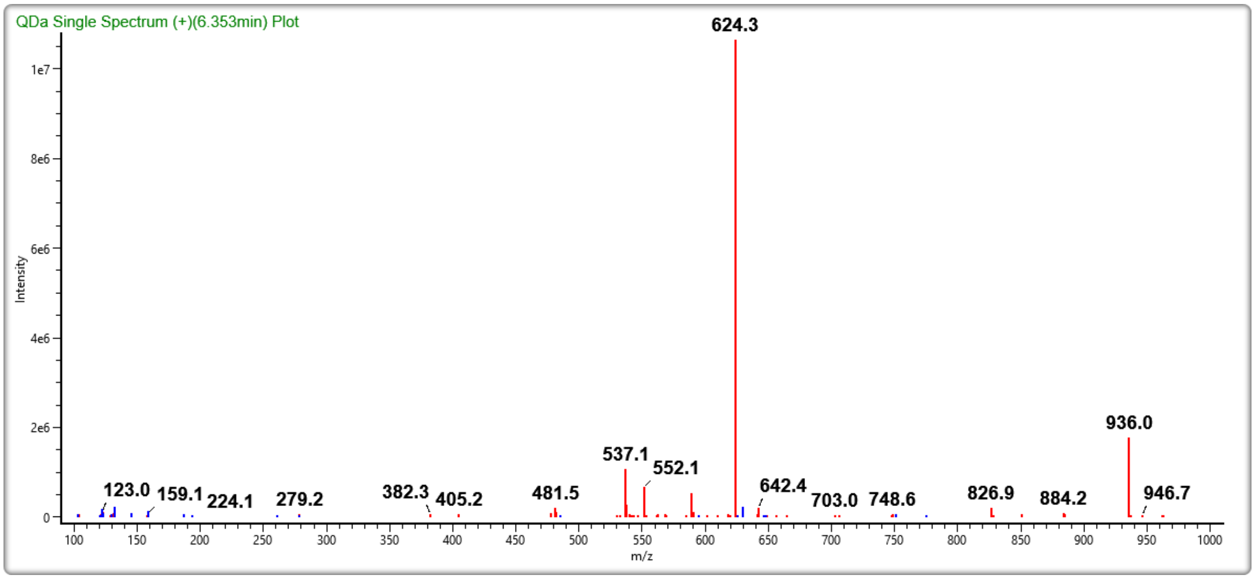


**Figure S10**. The characterization of PEP. HPLC chromatogram (absorbance at 214 nm) (top) and ESI-MS spectrum (bottom)

Fig. 2C and Fig. S1D

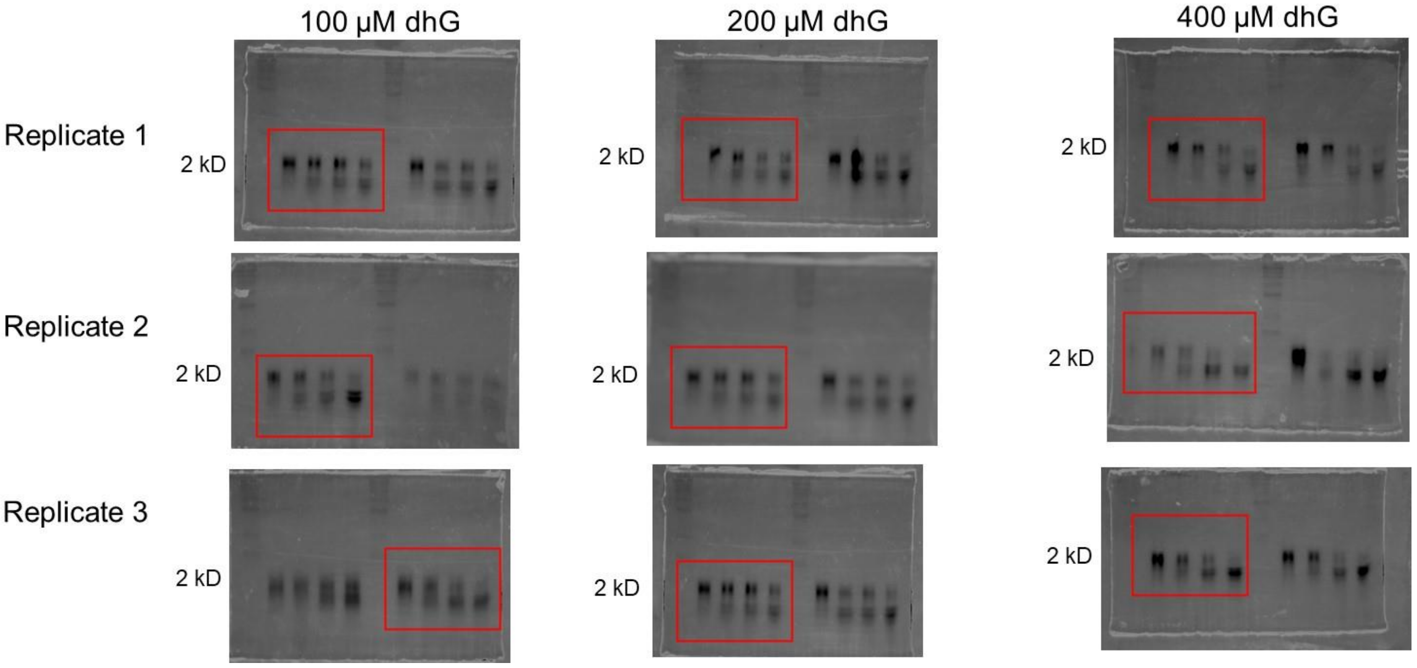


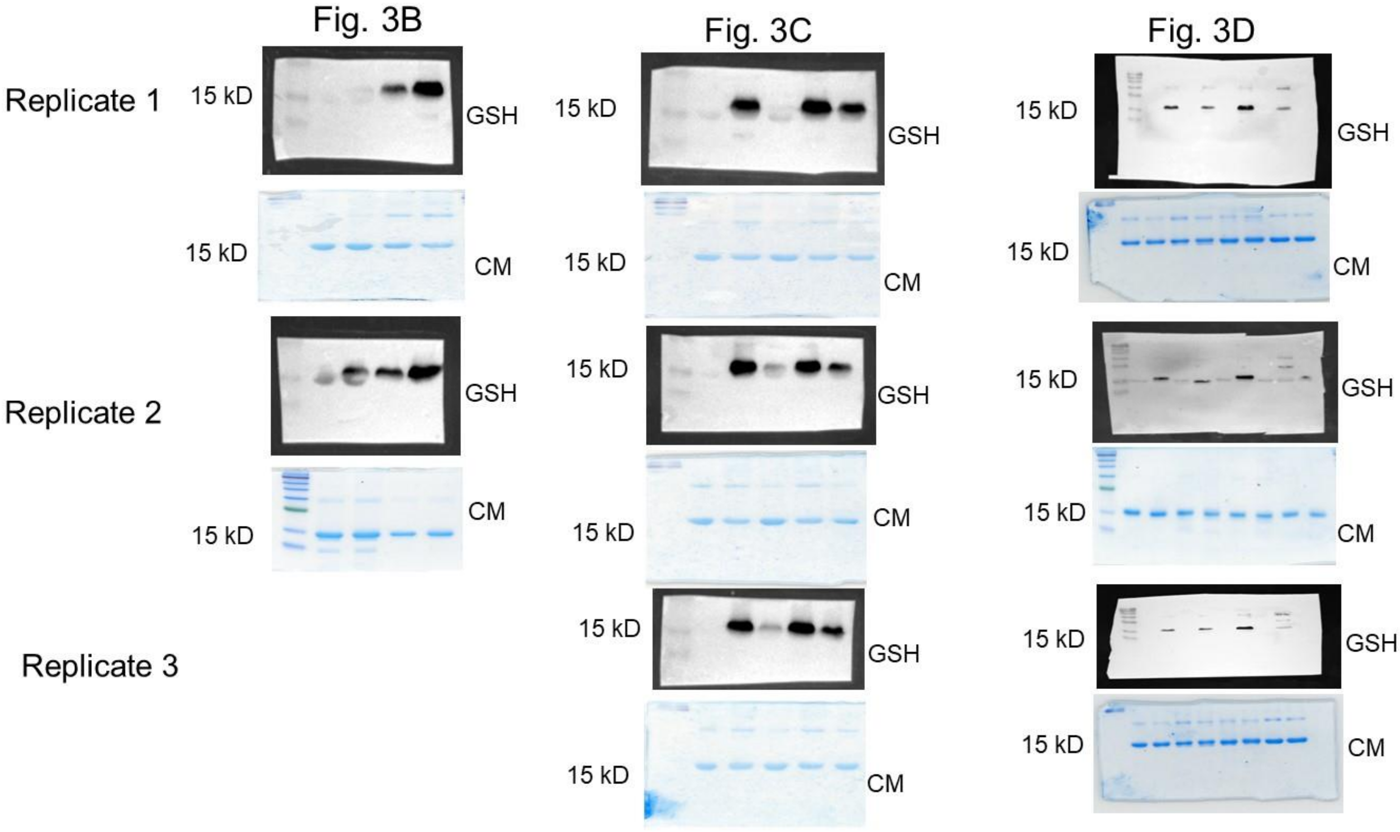

## Fig. 5A

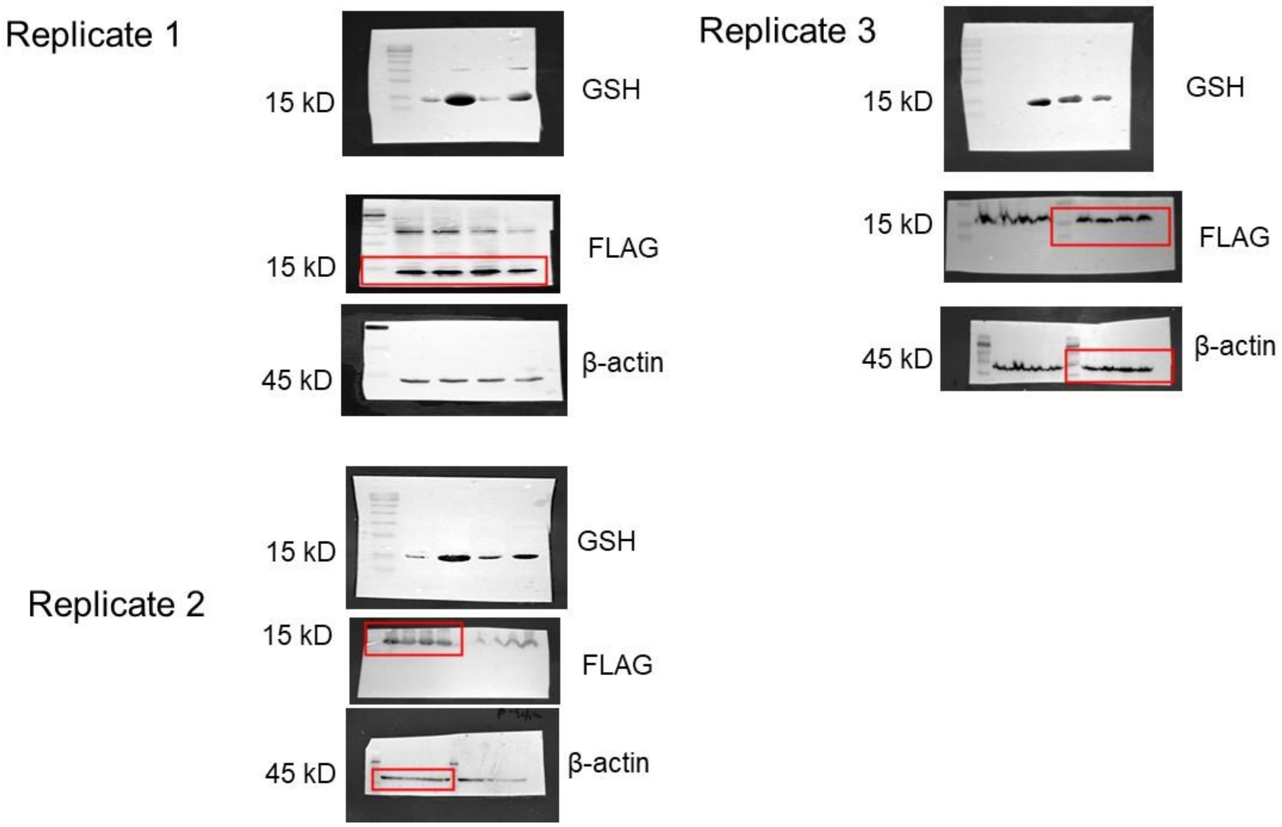

Replicate 1
15 kD
GSH
15 kD
FLAG
45 kD
β-actin
Replicate 2
15 kD
GSH
15 kD
FLAG
45 kD
β-actin
Replicate 3
15 kD
GSH
15 kD
FLAG
45 kD
β-actin


## Fig. 5C

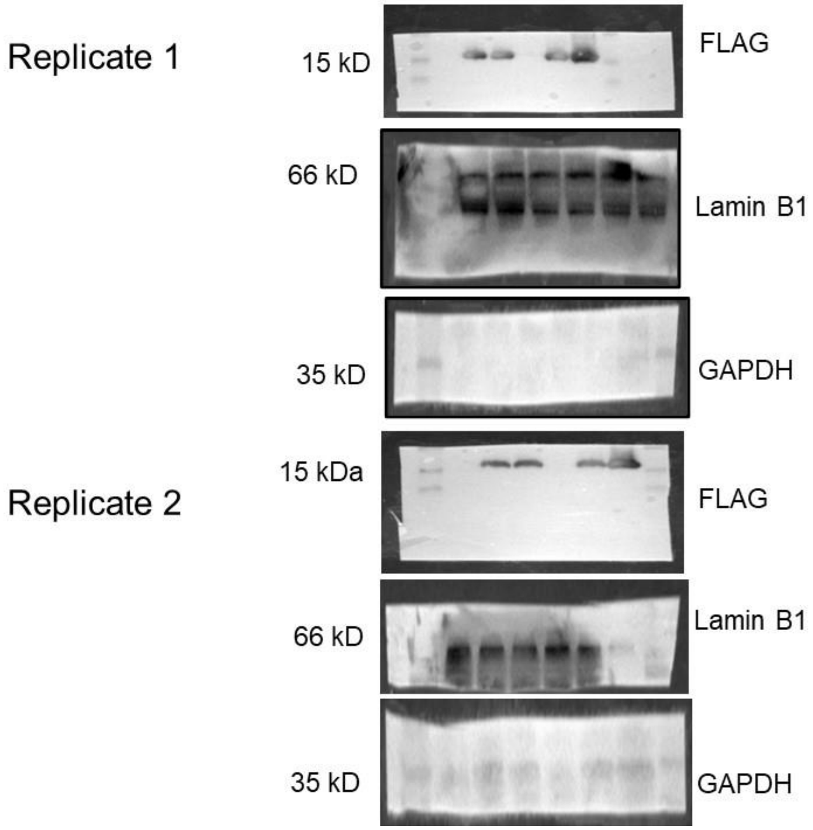

Replicate 1
15 kD
FLAG
66 kD
Lamin B1
35 kD
GAPDH
Replicate 2
15 kDa
FLAG
66 kD
Lamin B1
35 kD
GAPDH

Fig. S2A

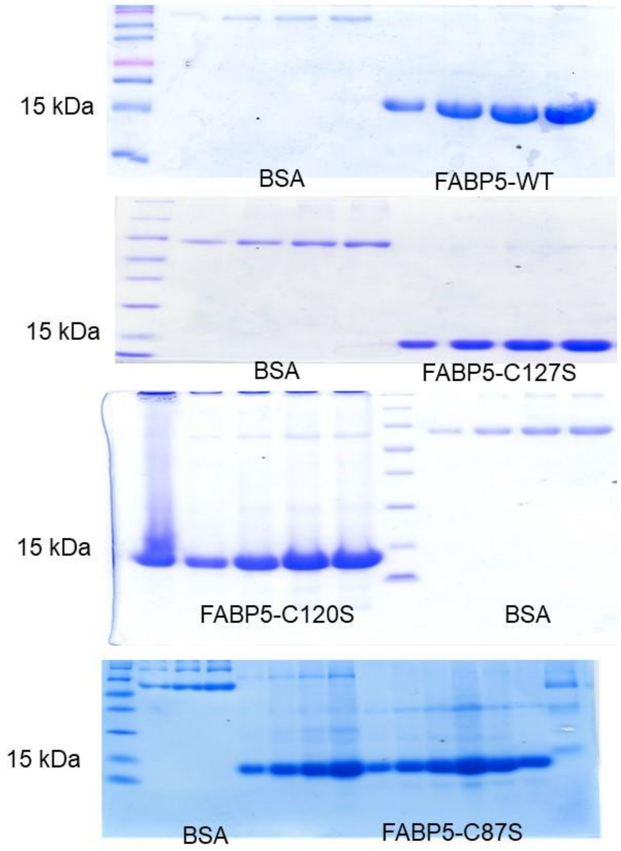


Fig. S3A

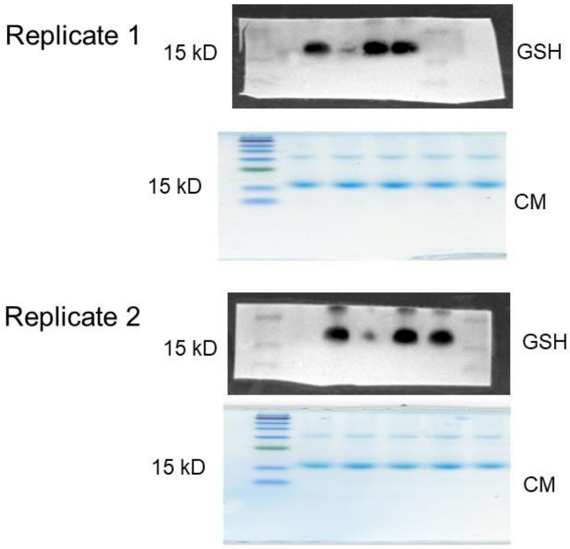


Fig. S3C

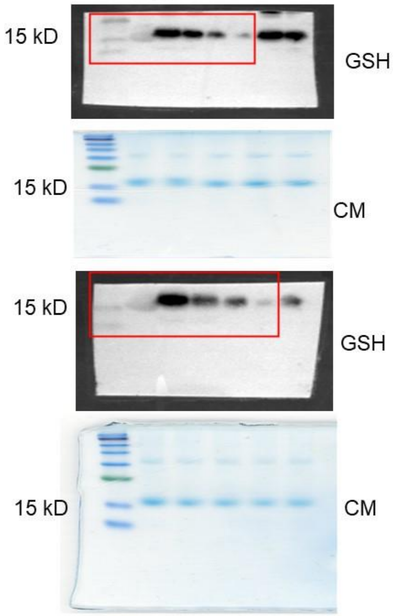


**Figure S11**. Raw images of gel blots in the manuscript.

## Experimental Methods

**Synthesis of dehydroglutathione (dhG)**. dhG was synthesized through the elimination of 2,4-dinitrothiophenolate from S-(2,4-dinitrophenyl)glutathione (GS-DNP) as described by Carthew *et al*.[S1] GS-DNP was synthesized with minor modification of the method of Patchornik *et al*.[S2] A solution of 1-chloro-2,4-dinitrobenzene (1.01 g, 5 mmol) in 10 mL of methanol was added dropwise to a solution containing glutathione (1.53 g, 5 mmol) dissolved in 1 N $NaHCO_3$ (20 mL). The solution was then stirred at room temperature for one hour, filtered, and the product was precipitated by the addition of 1 M HCl. The yellow precipitate was collected by vacuum filtration and dried to produce GS-DNP, which was used without further purification. GS-DNP (1 g) was dissolved in 0.5 M NaOH (50 mL), and the solution was stirred at room temperature for 30 min. The solution was then extracted four times with butanol (20 mL each). The organic layers were discarded, and the aqueous layer was mixed with Amberlite IRC-50 ion exchange resin (hydrogen form) (3 g) to remove sodium ions. The pH of the solution dropped from 12.4 to ~ 4.0. The aqueous solution was lyophilized, and a light brown solid was collected, yielding dhG (78%). The obtained product was recrystallized from ethanol. $^1$H-NMR spectrum is identical to the one reported.[S1] $^1$H-NMR (500 MHz, $D_2O$) δ 5.60 (s, 1H), 5.56 (s, 1H), 3.68 (s, 2H), 3.64 - 3.62 (m, 1H), 2.45 - 2.42 (m, 2H), 2.04 - 2.00 (m, 2H).

**dhG reaction with NAC**. To a solution of N-acetyl cysteine (0.036 mmol, 5.9 mg) in 1X phosphate-buffered saline (PBS) buffer (1 mL) was added dhG (0.036 mmol, 10 mg). The pH of the solution was adjusted to 8 using 1N $NH_4OH$ and stirred overnight at room temperature. The filtered aqueous solution was injected and separated on a reverse-phase C18 column (SunFire, Waters) in HPLC (Acquity, Waters) with a gradient elution of water and acetonitrile (5-95% gradient for 35 min). After lyophilization, the product was obtained as a white solid (5.5 mg, 35 % yield). The purity and mass were confirmed by ESI-MS and LC-MS. Calculated mass: 437.13 (M+H); Found mass: ESI m/z 437.30 (M+H). The adduct was also confirmed by $^1$H NMR. $^1$H-NMR (500 MHz, $D_2O$) δ 4.44 - 4.41 (m, 2H), 3.86 – 3.81 (m, 3H), 2.97 – 2.90 (m, 2H), 2.83 - 2.72 (m, 2H), 2.41 – 2.38 (m, 2H), 2.07 – 2.02 (m, 2H), 1.88 (s, 3H).

**Peptide synthesis**. Chloro-trityl chloride (0.2 g, 0.22 mmol) resin was suspended in 3 mL dichloromethane (DCM) for 0.3 h. Fmoc-Glu(O$^t$Bu)-OH•$H_2O$ (0.374 g, 0.88 mmol) and 5% DIPEA in 4 mL DMF were added to the resin. The mixture was stirred gently at room temperature for 3 h. The resin was washed sequentially with 3 x 2 mL DMF, 3 x 2 mL DCM, and 3 x 2 mL DMF. The resin was subjected to Fmoc deprotection and the amino acid coupling sequentially, following the Fmoc solid-phase peptide synthesis protocol. 20% piperidine in 3.5 mL DMF for 0.75 h was used for Fmoc deprotection. The amino acid (0.88 mm) was used with HATU (0.327 g, 0.85 mmol) and 5% DIPEA in 4.4 mL DMF with 3 h incubation. After the last coupling and Fmoc deprotection, the peptide was cleaved by incubating the resin in 95% TFA, 2.5 % TIPS, and 2.5% water for 3 h. 3 mL of cold diethyl ether was added to the filtrate. The peptide pellet was collected by centrifugation at 4,000 rpm for 2 min and redissolved in distilled water. The peptide was purified on a reverse-phase C18 column (SunFire, Waters) in HPLC (Acquity, Waters) with a gradient elution of water and acetonitrile (5-95% gradient for 35 min) and analyzed by ESI-MS: calculated mass, 624.0 $(M+3H)^{3+}$; found mass, 624.4 $(M+3H)^{3+}$.

**FAM-conjugation to peptide**. Fmoc-deprotected peptide synthesized on the resin was mixed with 3 eq. DIPEA and 5 eq. NHS-fluorescein in 1 mL DMSO. The reaction mixture was rotated for 5 h at room temperature in the dark. The reaction was quenched with 20 mM Tris-HCl buffer, pH 8.0. The resin was washed with 3 x 1 mL DMF, 3 x 1 mL DCM, and 3 x 1 mL DMF. The peptide was cleaved in 95% TFA, 2.5 % TIPS, and 2.5% water, purified, and analyzed as described in the peptide synthesis: calculated mass, 743.3 $(M+3H)^{3+}$; found mass, 743.4 $(M+3H)^{3+}$.

**dhG reaction with Cys-containing peptides**. 1 mM dhG was added to 0.1 mM of Cys-containing peptide (PEP) in PBS, pH 8.0, with 10 μM DTT. The mixture was incubated at room temperature for 1 h. The reaction mixture was diluted with Milli Q water containing 0.1 % formic acid. The formation of dhG modification on the peptide was analyzed by HPLC-MS (Acquity, Waters) on a reverse-phase C18

column (SunFire, Waters) with a gradient elution of water and acetonitrile (5-95% gradient for 15 min). For gel-based kinetic analysis, 10 μM FAM-PEP was prepared in PBS containing 10 μM DTT. 100, 200, and 400 μM dhG were added and mixed, with a final pH of 8.0. Reactions were incubated at room temperature for 5, 15, 30, and 60 min. Reactions were quenched using 2x bromophenol blue loading dye containing 1 mM DTT and analyzed on neutral urea-based gel electrophoresis.

**Protein purification**. pET2a-FABP5 wild-type plasmid was a gift from Dr. Martin Kaczocha at Stony Brook University. FABP5 mutants (C127S, C120S, and C87S) were generated using site-directed mutagenesis (C87S forward primer: 5'-GCAGAAAAACTCAGACTGTCAGCAACTTTACAGATGGTG-3' and reverse primer: 5'-CACCATCTGTAAAGTTGCTGACAGTCTGAGTTTTTCTGC-3'; C120S forward primer: 5'-GAAATTAGTGGTGGAGAGCGTCATGAACAATGTCACC-3' and reverse primer: 5'-GGTGACATTGTTCATGACGCTCTCCACCACTAATTTC-3'; C127S forward primer: 5'-GTCATGAACAATGTCACCAGCACTCGGATCTATGAAAAAG-3' and reverse primer: 5'-CTTTTTCATAGATCCGAGTGCTGGTGACATTGTTCATGAC-3'). FLAG tag was inserted into FABP5 constructs to generate pET2a-FLAG-FABP5 plasmids using inverse-PCR (forward primer: 5'-GATGACGACAAGCATATGGCCACAGTTCAGCAGCTG-3' and reverse primer: 5'-GTCTTTGTAGTCCCCGCTGCCGCGCGGCAC-3'). FABP5 plasmids were transformed into BL21 (DE3) competent cells. A single colony was inoculated into 5 mL Luria Broth (LB) medium containing 50 μg/mL kanamycin and grown at 37 ºC for 16 h. 2.5 mL overnight culture was inoculated into 1 L LB medium containing 50 μg/ml kanamycin and grown at 37 ºC until $OD_{600}$ reached 0.5. Protein expression was induced with 0.5 mM isopropyl β-D-1-thiogalactopyranoside (IPTG) and incubated at 18 ºC for 18 h. Cells were pelleted by centrifugation at 5,000 rpm and washed with cold Tris-HCl buffer. Cells were lysed using a French press with a lysis buffer (25 mM Tris, pH 7.4, 300 mM NaCl, 1 mM DTT, and Pierce protease inhibitor, pH 7.4). Clarified lysate was incubated with Ni-NTA agarose beads at 4 ºC for 2 h. Proteins were eluted using elution buffer (25 mM Tris, 75 mM NaCl, 300 mM imidazole, 1 mM

DTT, pH 7.4). Pure fractions were combined and dialyzed in dialysis buffer (25 mM Tris, pH 7.4, 75 mM sodium chloride, 1 mM DTT, and 5% glycerol).

**Protein delipidation.** 100 mg of lipophilic Sephadex LH-20-100 (hydroxypropyl dextran with C13-C18 alkyne ethers, dry beads)[S3] (Sigma, H6383) was activated by immersing in 500 µL of buffer (10 mM potassium phosphate, 150 mM potassium chloride, 0.2 g/L sodium azide, pH 7.4) in a 1.5 mL microcentrifuge tube, followed by shaking at 37 °C for 2.5 h. 500 µL of FABP5 (2 mg/mL) was mixed with the bead suspension. The protein bead mixture was shaken at 37 °C for 2 h. The beads were separated from the protein using a 0.22-µm Millipore Amicon filter. Two cycles of the treatment were carried out. Delipidated FABP5 was dialyzed in PBS (10 mM $Na_2HPO_4$, 1.8 mM $KH_2PO_4$, 2.7 mM KCl, 137 mM sodium chloride, 1 mM DTT, and 5% glycerol, pH 7.4). Lipid removal was confirmed by staining delipidated protein with iodine solution (0.5 mg $I_2$ in 1 mL chloroform) on a TLC plate.

**FABP5 dhG modification or S-glutathionylation**. To FABP5 protein (0.5 mg) in PBS (pH 8.0) (0.5 mL) containing 1 mM DTT was added 10 mM dhG or 5 mM GSSG. The reaction was incubated at room temperature for 12 h unless stated otherwise. Unreacted dhG and GSSG were removed through dialysis. dhG modification or S-glutathionylation was analyzed using Coomassie stain or western blot using glutathione antibody. To measure dhG modification in the reducing condition, FABP5 modified by dhG or GSSG was incubated with 20 mM DTT for 3 h at room temperature before western blot analysis. To measure the reversibility of FABP5 S-glutathionylation over time, FABP5 incubated with 5 mM GSSG was run through the gel filtration column (Cytiva NAP-10). Purified FABP5 sample was collected at different time points and analyzed by western blot with glutathione antibody.

**Mass spectrometry.** For MALDI-TOF analysis, 50 µg of FABP5-SG or FABP5-SSG was dialyzed into Milli-Q water containing 0.1% TFA. 1 µL sample was mixed with 1 µL of 2-cyano-3-(4-hydroxyphenyl)-2-propenoic acid) (α-CHCA) matrix solution (10 mg α-CHCA in 1 mL methanol with 0.1% TFA). 1 µL

sample was spotted on a MALDI plate and analyzed by MALDI-TOF (Bruker). For MS/MS analysis, 50 μg of FABP5-SG was treated with CNBr (10 μL of a solution prepared by 10 mg CNBr in 0.5 mL of 0.5 N HCl). The sample was incubated for 12 h at room temperature. The sample was purified on a C18 ziptip, lyophilized, and resuspended at a concentration of 1 μg/μL in 0.1% trifluoroacetic acid with sonication. LC-MS/MS analysis was carried out with an Ultimate 3000 RSLCnano system (Thermo Scientific) coupled to an Orbitrap Eclipse mass spectrometer (Thermo Scientific). For the chromatographic separation, the column compartment temperature was set to 45 °C, and the flow rate was set to 0.200 μL/min. Buffer A consisted of 0.1% formic acid in Milli-Q water, and Buffer B consisted of 0.1% formic acid in acetonitrile. 1 μg of the sample was loaded onto a trap column (nanoEase M/Z Symmetry C18, 100 Å, 120 μm x 20 mm, Waters) and washed for 10 min with Buffer A. The trap column was placed in line with the resolving column (nanoEase M/Z Peptide BEH C18, 130 Å, 75 μm x 250 mm, Waters) and separated using a linear gradient increasing from 5% to 30% B over 75 min, then from 30% to 80% B over 5 min, held at 80% B for 10 min at an increased flow rate of 0.300 μL/min, and equilibrated at 5% B for 10 min.

MS data were acquired in data-dependent acquisition mode using a cycle time of 3 s. For each cycle, the MS1 scan was performed in the Orbitrap at 120k resolution, and precursor ions with a charge state between 2+ and 8+ and a mass of 350 – 1800 m/z were isolated (1.4 m/z window) for fragmentation by higher-energy collisional dissociation (30%). MS2 scans were performed in the ion trap with a scan range of 200 – 1400 m/z, a maximum injection time of 35 ms, and the scan rate was set to Turbo mode. Dynamic exclusion was enabled at 20 s.

RAW files were searched with MaxQuant 2.6.5.0 against the UniProt human complete database downloaded 2024.10.05 (83,413 entries) plus a contaminant database. N-terminal acetylation and methionine oxidation were variable modifications. dhG addition to cysteine ($C_{10}H_{15}N_3O_6$, 273.09608) was used for the modification. All other parameters were left at default values. Peptide spectra matches were accepted at a 1% false discovery rate as determined by a reverse database search. Peptide modification was analyzed using Skyline software (version 24.1.0.199). dhG modification ($C_{10}H_{15}N_3O_6$,

273.09608) was input into the structural modification tab. All other parameters were left to default values. Peptide libraries were built by importing all msms.txt files into Skyline using a default spectral library cut-off score of 0.95. Raw files were imported to Skyline for peak picking. Peptides that showed dhG mass modification were identified.

**Isothermal titration calorimetry**. FABP5 was dialyzed into 25 mM Tris-HCl and 75 mM NaCl pH 7.4. The affinity of linoleic acid for FABP5 was measured in MicroCal ITC (Malvern). The syringe was filled with linoleic acid (1 mM in Tris-HCl buffer). The sample cell was filled with FABP5 (0.1 mM in Tris-HCl buffer). The reference cell was filled with Tris-HCl buffer. The sample cell and the syringe were first equilibrated to 25 °C, followed by an initial delay of 60 seconds before the first injection of 0.4 μL of linoleic acid into the sample cell for 4 seconds. For the remaining injections, a 2 μL volume of linoleic acid solution was injected into the sample cell for 4 seconds. The syringe was stirred continuously in the sample cell at 750 rpm. A total of 19 injections were performed with a spacing of 150 seconds. The binding data were analyzed using the MicroCal ITC software. A one-site binding model was fitted to the data to calculate the binding parameters. The equilibrium dissociation constant ($K_D$), Gibbs energy ($\Delta G$), enthalpy ($\Delta H$), and entropy ($T\Delta S$) were determined from the curve.

**Cell culture**. MCF-7 cells were purchased from ATCC and maintained in EMEM medium supplemented with 10 % fetal bovine serum (FBS, Hyclone, Cytiva), penicillin (100 units/mL), and streptomycin (100 μg/mL) (Pen-Strep). Cells were maintained at 37 °C in 5 % $CO_2$ in a humid atmosphere.

**Fusogenic liposome preparation**. Stock solutions of 1,2-dioleoyl-sn-glycero-3-phosphoethanolamine (DOPE), *N, N, N*-trimethyl-2,3-bis(oleoyloxy)propan-1-aminium methylsulfate (DOTAP) and 1,1'-dioctadecyl-3, 3, 3', 3'-tetramethylindotricarbocyanineiodide (DiR') were prepared in chloroform with a ratio of 1:1:0.1 (w/w/w). Chloroform was removed under vacuum. 60 μg dried lipids were resuspended in PBS (0.15 mL), pH 7.4, containing 12 μg FABP5 construct. The suspension was mixed thoroughly

and sonicated for 5 min on ice. The suspension was passed through the Whatman Nuclepore Track-Etch membrane with 0.1 μm pore size using an AVANTI Polar Lipids extruder. Liposomes with encapsulated proteins were purified via gel filtration with NAP5 columns (Cytiva).

**Protein delivery**. Cells in a 10 cm dish were washed with 1xPBS. EMEM medium was added and incubated at 37 °C for 30 mins. 0.15 mL of PBS solutions containing fusogenic liposomes without or with FABP5 construct were diluted with 0.15 mL EMEM. 0.15 mL of the diluted solutions were added to cells. Cells were incubated at room temperature or 37 °C for 1 h. Cells were washed with 1xPBS (1 mL x 4). Cells were lysed for western blot to confirm delivery. For migration assay, EMEM medium (without FBS and Pen-Strep) was added, followed by 50 μM linoleic acid. Cells were incubated at 37 °C for 24 h.

**Western blot**. Cells were lysed in ice-cold RIPA buffer (300 μL per 10 cm dish). After incubation at 4 °C for 30 min and centrifugation at 16,000 x g for 10 min at 4 °C, the supernatant was collected. The protein concentration was determined by Bradford assay. Proteins or lysates were separated by SDS-PAGE (12%) and transferred to the PVDF membrane. The membrane was blocked with 5 % BSA in Tris-buffered saline with 0.1% tween (TBST) and incubated with the following primary antibodies: FLAG (Sigma-Aldrich, F1804, 1:1000), glutathione (Virogen, 101-A-250, 1:1000), β-actin (Santa Cruz Biotechnology, sc-8432, 1:1000), GAPDH (Cell signaling, D4C6R, 1: 1000), and Lamin B1 (Cell signaling, D4Q4Z, 1: 1000). The membrane was then incubated with horseradish peroxidase (HRP)-conjugated anti-mouse IgG (NA931V, Sigma, 1:1000) and anti-rabbit IgG, (NA934V, Sigma, 1:2000) to visualize the proteins by chemiluminescence (SuperSignal West Pico). Blots were imaged using the iBright imaging system (Thermo Scientific).

**Cytoplasmic and nuclear extraction**. Cytoplasmic and nuclear proteins were extracted using NE-PER™ Nuclear and Cytoplasmic Extraction Reagents (Thermo Scientific, 78833). Cells were harvested

with trypsin-EDTA and centrifuged at 500 × g for 5 min. Cells were washed with 1xPBS and pelleted by centrifugation at 500 × g for 3 min. The cell pellet was dried and mixed with 500 μL of ice-cold cytoplasmic extraction reagent I (CER I), followed by 27.5 μL of ice-cold cytoplasmic extraction reagent II (CER II). After incubating for 1 min, the sample was centrifuged for 5 min at 16,000 × g. The supernatant was collected as cytoplasmic extracts. The insoluble (pellet) fraction was suspended and lysed in 250 μL ice-cold nuclear extraction reagent (NER). After centrifugation at 16,000 × g for 10 minutes, the supernatant was collected as nuclear extracts.

**PPARβ/δ activation**. The method was adapted from prior work.[S4] PPARβ/δ transcriptional activation was performed using the transcriptional factor assay kit (Abcam, ab133106). After protein delivery, the MCF7 nuclear extracts were prepared and mixed with the complete transcription factor binding assay buffer (CTFB), which was added to a 96-well plate immobilized with peroxisome proliferator response element (PPRE) and incubated at 4 °C. After washing with wash buffer (x5), each well in the plate was incubated with PPARβ/δ antibody (Abcam, AB133106, 1:1000) in binding buffer for 1 h at room temperature. After washing with wash buffer (x5), anti-rabbit HRP conjugate (Abcam, AB133106) was incubated for 1 h at room temperature. After incubating developing and stop solutions (colorimetric measurement), the bound PPARβ/δ was quantified by reading absorbance at 450 nm using a microplate reader (BioTek plate reader).

**Confocal microscopy**. After delivery of FABP5 constructs using fusogenic liposomes for 1 h, cells were washed with PBS. 60 μM linoleic acid was added to cells and incubated for 1 h at room temperature. Cells were washed with PBS and fixed with 4 % paraformaldehyde for 15 min. Cells were permeabilized with 0.1 % Triton X-100 for 15 min. Cells were blocked with 2% BSA for 30 min at room temperature. Cells were incubated with primary antibodies: FLAG (Sigma, F1804, 1:100) for 12 h at 4 °C. Cells were washed with PBS and incubated with Alexa Fluor™ 488-conjugated goat anti-mouse IgG (H+L) (Invitrogen, A11001, 1:200) for 1 h at room temperature. The nuclei were stained with DAPI

(Invitrogen, P36931). Cells were imaged using the confocal microscope LSM 700 (Zeiss). The localizations of FLAG-FABP5 constructs in the nucleus and cytoplasm in the images were quantified using ImageJ software. Briefly, the nuclei channel (blue) was used to select the boundary of the nucleus (by adjusting the threshold of the nuclei channel with a black background), which was saved as an ROI extension. The nuclei ROI file was selected in the green channels to separate the nuclei and cytoplasmic regions. The fluorescence intensities within and outside the nucleus boundary were measured to show the localizations of FABP5 in the nucleus over the cytoplasm. The data were analyzed using GraphPad Prism.

For the localization analysis in the cytoplasm and endosome, after incubating fusogenic liposomes for 1 h, cells were washed with PBS and fixed with 4 % paraformaldehyde for 15 min. Cells were permeabilized with 0.1 % Triton X-100 for 15 min. Cells were blocked with 2% BSA for 30 min at room temperature. Cells were incubated with primary antibodies: FLAG (Sigma, F1804, 1:100) and RAB9A (Cell Signaling, D52G8, 1:100) for 12 h at 4 °C. Cells were washed with PBS and incubated with Alexa Fluor$^{TM}$ 488-conjugated goat anti-mouse IgG (H+L) (Invitrogen, A11001, 1:200) and Alexa Fluor$^{TM}$ 647-conjugated goat anti-rabbit IgG (H+L) (Invitrogen, A21245, 1:200) for 1 h at room temperature. The nuclei were stained with DAPI (Invitrogen, P36931). Cells were imaged using the confocal microscope LSM 700 (Zeiss). For quantification, the numbers of pixels showing only green and both green/red were counted to determine FABP5 in the cytoplasm and endosome, respectively. Images were analyzed by ImageJ (n=10 images).

**Migration assay.** MCF-7 cells were seeded into 12 well plates coated with fibronectin/gelatin to produce a fully confluent monolayer (1.4 x $10^5$ cells). After overnight, the monolayer cells were scratched using a 10 µL pipet tip to create an even wound. Cells were washed with warm PBS (1 mL x 4) to remove non-adherent cells and incubated in EMEM for 30 min. 50 µL of fusogenic liposome diluted with 50 µL EMEM medium was added to cells, followed by incubating at 37 °C for 1 h. After washing with PBS (1 mL x 4), 50 µM linoleic acid was added to cells in EMEM, and cells were incubated at

37 °C. The wound area was imaged at 0 and 24 h using a light microscope connected to a camera. Images were analyzed using an MRI wound healing tool in ImageJ software.

**NUT gel electrophoresis**. Neutral pH urea Triton-polyacrylamide gel electrophoresis (NUT gel) was prepared according to the prior work.[S5] NUT gel (15% resolving) was prepared and run with NUT-PAGE running buffer (22 mM MOPS pH 7.0, 100 mM imidazole). The samples were run at 125 V for 20 min, then at 100 V for 12 h at room temperature. The dhG-modified and non-modified peptides were visualized by fluorescence using the iBright imager (Thermo Scientific) and quantified using ImageJ software.

**Kinetic analysis of dhG modification**. Time-dependent samples at three different concentrations of dhG (100, 200, and 400 μM) were resolved on NUT-gels. The amounts of the peptide (starting peptide) remaining at different time points were quantified using ImageJ software. The logarithm values of the starting peptide at different times were plotted for reaction rates. Reactions were assumed to follow a pseudo-first-order kinetic mechanism. Reaction rates were plotted as a function of dhG concentrations. The second-order rate constant of dhG was obtained from the linear curve.

**Statistical analysis**. All data are shown with the means ± SD and analyzed by one-way or two-way ANOVA followed by Tukey's *post-hoc* test. The value $p < 0.03$ is statistically significant.

**Supplementary Reference**


S1. R. S. Asquith and P. Carthew, *Biochim Biophys Acta*, 1972, **285**, 346-351.
S2. M. Sokolovsky, T Sadeh and A. Patchornik, *J Am Chem Soc* 1963, **86**, 1212-1217.
S3. Q. Wang, S. Rizk, C. Bernard, M. P. Lai, D. Kam, J. Storch and R. E. Stark, *Biochem Biophys Rep*, 2017, **10**, 318-324.
S4. C. Mori, J. Y. Lee, M. Tokumoto and M. Satoh, *Int J Mol Sci*, 2022, **23**.
S5. C. J. Buehl, X. Deng, M. Liu, M. J. McAndrew, S. Hovde, X. Xu and M. H. Kuo, *Biotechniques*, 2014, **57**, 72-80.
